# Multivariate Tail Coefficients: Properties and Estimation

**DOI:** 10.3390/e22070728

**Published:** 2020-06-30

**Authors:** Irène Gijbels, Vojtěch Kika, Marek Omelka

**Affiliations:** 1 Department of Mathematics and Leuven Statistics Research Center (LStat), KU Leuven, 3001 Leuven, Belgium; vojtech.kika@kuleuven.be; 2 Department of Probability and Mathematical Statistics, Faculty of Mathematics and Physics, Charles University, 186 75 Prague, Czech Republic; omelka@karlin.mff.cuni.cz

**Keywords:** archimedean copula, consistency, estimation, extreme-value copula, tail dependency, multivariate analysis, Primary: 60Exx, Secondary: 62H20, 62G32

## Abstract

Multivariate tail coefficients are an important tool when investigating dependencies between extreme events for different components of a random vector. Although bivariate tail coefficients are well-studied, this is, to a lesser extent, the case for multivariate tail coefficients. This paper contributes to this research area by (i) providing a thorough study of properties of existing multivariate tail coefficients in the light of a set of desirable properties; (ii) proposing some new multivariate tail measurements; (iii) dealing with estimation of the discussed coefficients and establishing asymptotic consistency; and, (iv) studying the behavior of tail measurements with increasing dimension of the random vector. A set of illustrative examples is given, and practical use of the tail measurements is demonstrated in a data analysis with a focus on dependencies between stocks that are part of the EURO STOXX 50 market index.

## 1. Introduction

Assume that we have a *d*-variate random vector and we are interested in the tendency of the components to achieve extreme values simultaneously, which is taking extremely small or extremely large values. In the bivariate setting, when d=2, this so-called tail dependence has been studied thoroughly in the literature. Bivariate lower and upper tail coefficients appeared for example in [1] but the idea of studying bivariate extremes dates back to [2]. These coefficients, being conditional probabilities of an extreme event given that another event is also extreme, have become the standard tool to quantify tail dependence of a bivariate random vector. Later, a generalization into arbitrary dimension *d* became of interest. The presence of more than two components however brings difficulties of defining tail dependency and several proposals appeared in the literature. These proposals include those made by [3,4] or [5] who adopted different strategies for conditioning in general dimensions. Further proposals were made for specific copula families, for example, by [6] for Archimedean copulas or by [7] for extreme-value copulas.

In this paper, we aim to contribute to the discussion on the appropriateness of multivariate tail coefficients, from the view point of properties that one would desire such coefficients to have. This study also entails the proposal of some new multivariate tail measures, for which we establish the properties. We investigate an estimation of the discussed multivariate tail coefficients and establish consistency of all estimators. It is also of particular interest to find out how tail dependence measures behave when the dimension *d* increases.

The organization of the paper is as follows. In Section 2, we briefly review some basic concepts about copulas and classes of copulas that will be needed in subsequent sections. Section 3 is devoted to the study of various multivariate tail dependence measures, whereas Section 7 discusses statistical estimation of these measures, including consistency properties. Section 4 investigates some further probabilistic properties of the multivariate tail dependence measures. Section 5 studies the behavior of the tail coefficient measures for Archimedean copulas when the dimension increases to infinity. A variety of illustrative examples is provided in Section 6, and it accompanies the studies that are presented in Section 3 and Section 5. Finally, in Section 8, it is demonstrated how multivariate tail coefficients contribute in getting insights into dependencies between stocks that are part of the EURO STOXX 50 market index.

## 2. Multivariate Copulas

In this section, we briefly introduce concepts and notation from copula theory that will be necessary in the rest of this text. For more details on copulas, see e.g., [8].

### 2.1. Basic Properties. Survival and Marginal Copulas

Suppose that we have a *d*-variate random vector $X={\left(X_{1},\cdots ,X_{d}\right)}^{⊤}$ having a joint distribution function *F*. Let further $F_{j}$ denote the continuous marginal distribution function of $X_{j}$ for j=1,⋯,d. Sklar’s theorem [9] describes the relationship between the joint distribution function and the marginals that are given by a unique copula function $C_{d}:{\left[0,1\right]}^{d}\to \left[0,1\right]$ such that

$$\begin{array}{c} F\left(x_{1},\cdots ,x_{d}\right)=C_{d}\left(F_{1}\left(x_{1}\right),\cdots ,F_{d}\left(x_{d}\right)\right),\;{\left(x_{1},\cdots ,x_{d}\right)}^{⊤}\in R^{d}. \end{array}$$

We denote the set of all *d*-variate copulas by Cop(d). From the above relationship, it is easily seen that the random vector $U={\left(U_{1},\cdots U_{d}\right)}^{⊤}={\left(F_{1}\left(X_{1}\right),\cdots ,F_{d}\left(X_{d}\right)\right)}^{⊤}$ has a joint distribution function $C_{d}$, that is, with $u={\left(u_{1},\ldots ,u_{d}\right)}^{⊤}\in {\left[0,1\right]}^{d}$, $C_{d}\left(u\right)=P\left(U\leq u\right)$. The inequalities of vectors in this text are understood component-wise.

The survival function ${\bar{C}}_{d}$ that is associated to a copula $C_{d}$ is defined as ${\bar{C}}_{d}\left(u\right)=P\left(U>u\right)$. The survival copula $C_{d}^{S}$ that is associated to a copula $C_{d}$ is defined as the copula of the random vector 1−U, that is

(1) $$\begin{array}{c} C_{d}^{S}\left(u\right)=P\left(1-U\leq u\right)={\bar{C}}_{d}\left(1-u\right). \end{array}$$

Let π be a permutation of the set of indices {1,⋯,d}, i.e., π:{1,⋯,d}→{1,⋯,d}. The copula $C_{d}^{\pi }$ is defined using a copula $C_{d}$ as [10]

$$C_{d}^{\pi }\left(u_{1},\cdots ,u_{d}\right)=C_{d}\left(u_{\pi (1)},\cdots ,u_{\pi (d)}\right),\;\forall u\in {\left[0,1\right]}^{d}.$$

In every point of the unit hypercube ${\left[0,1\right]}^{d}$, the value of a copula $C_{d}$ is restricted by the lower Fréchet’s bound $W_{d}\left(u\right)=\max\left(\sum_{j=1}^{d}u_{j}-d+1,0\right)$ and the upper Fréchet’s bound $M_{d}\left(u\right)=\min\left(u_{1},\cdots ,u_{d}\right)$. In other words,

$$\begin{array}{c} W_{d}\left(u\right)\leq C_{d}\left(u\right)\leq M_{d}\left(u\right),\;\forall u\in {\left[0,1\right]}^{d}. \end{array}$$

The function $M_{d}$ is a copula for any d≥2 and it is often called the comonotonicity copula, since it corresponds to the copula of a random vector X whose arbitrary component can be expressed as a strictly increasing function of any other component. If the components of a random vector X are mutually independent, the copula of X is the independence copula $\Pi_{d}\left(u\right)=\prod_{j=1}^{d}u_{j}$.

The copula that is associated to any subset of components of a *d*-dimensional random vector X is called a marginal copula of $C_{d}$. A marginal copula might be calculated from the original copula by setting arguments corresponding to the unconsidered components to 1. For example, the marginal copula $C_{d-1}^{\left(1,\cdots ,d-1\right)}$ of ${\left(X_{1},\cdots ,X_{d-1}\right)}^{⊤}$ can be obtained as

$$C_{d-1}^{\left(1,\cdots ,d-1\right)}\left(u_{1},\cdots ,\cdots u_{d-1}\right)=C_{d}\left(u_{1},\cdots ,u_{d-1},1\right),$$

where $C_{d}$ is the copula of X. Marginal copulas can be used to calculate the survival function ${\bar{C}}_{d}$ of a copula $C_{d}$, since

(2) $${\bar{C}}_{d}\left(u\right)=1+\sum_{j=1}^{d}{\left(-1\right)}^{j}\sum_{1\leq k_{1}<\cdots <k_{j}\leq d}C_{j}^{\left(k_{1},\cdots ,k_{j}\right)}\left(u_{k_{1}},\cdots ,u_{k_{j}}\right).$$

### 2.2. Classes of Archimedean and Extreme-Value Copulas

In the study here, we pay particular attention to two classes of copulas: multivariate extreme-value copulas and multivariate Archimedean copulas.

**Definition** **1.** *A d-variate copula $C_{d}$ is called an extreme-value copula if it satisfies*

$$\begin{array}{c} C_{d}\left(u_{1},\cdots ,u_{d}\right)={\left[C_{d}\left(u_{1}^{1/m},\cdots ,u_{d}^{1/m}\right)\right]}^{m} \end{array}$$

*for every integer m≥1 and $u\in {\left[0,1\right]}^{d}$.*

This definition is only one of many ways how to define extreme-value copulas. For other definitions and properties, see, for example, ref. [11]. Every extreme-value copula $C_{d}$ can be expressed in terms of a so-called stable tail dependence function $\ell_{d}:{\left[0,1\right)}^{d}\to \left[0,\infty \right)$ as

(3) $$\begin{array}{c} C_{d}\left(u_{1},\cdots ,u_{d}\right)=\exp\left(-\ell_{d}\left(-\log u_{1},\cdots ,-\log u_{d}\right)\right). \end{array}$$

Denote by $\Delta_{d-1}$ the *d*-dimensional unit simplex

$$\begin{array}{c} \Delta_{d-1}=\left\{\left(w_{1},\cdots ,w_{d}\right)\in {\left[0,\infty \right)}^{d}:w_{1}+\cdots +w_{d}=1\right\}. \end{array}$$

Every extreme-value copula can be equivalently expressed in terms of Pickands dependence function $A_{d}:\Delta_{d-1}\to \left[1/d,1\right]$ as

(4) $$\begin{array}{ll} C_{d}\left(u_{1},\cdots ,u_{d}\right) & =\exp\left[\left(\sum_{j=1}^{d}\log u_{j}\right)A_{d}\left(\frac{\log u_{1}}{\sum_{j=1}^{d}\log u_{j}},\cdots ,\frac{\log u_{d}}{\sum_{j=1}^{d}\log u_{j}}\right)\right]\\ & ={\left(\prod_{j=1}^{d}u_{j}\right)}^{A_{d}\left(\frac{\log u_{1}}{\sum_{j=1}^{d}\log u_{j}},\cdots ,\frac{\log u_{d}}{\sum_{j=1}^{d}\log u_{j}}\right)} \end{array}$$

The function $A_{d}$ is the restriction of the function $\ell_{d}$ on the unit simplex and given as

(5) $$\begin{array}{c} A_{d}\left(\frac{x_{1}}{\sum_{j=1}^{d}x_{j}},\cdots ,\frac{x_{d}}{\sum_{j=1}^{d}x_{j}}\right)=\frac{1}{x_{1}+\cdots +x_{d}}\ell_{d}\left(x_{1},\cdots ,x_{d}\right). \end{array}$$

Further, $A_{d}$ is convex and it satisfies $\max\left(w_{1},\cdots ,w_{d}\right)\leq A_{d}\left(w_{1},\cdots ,w_{d}\right)\leq 1$, for $w={\left(w_{1},\cdots ,w_{d}\right)}^{⊤}\in \Delta_{d-1}$. The comonotonicity copula $M_{d}$ and the independence copula $\Pi_{d}$ are both extreme-value copulas with respective Pickands dependence functions $A_{d}\left(w\right)=\max\left(w_{1},\cdots ,w_{d}\right)$ and $A_{d}\left(w\right)=1$, i.e., the lower and upper bounds above.

Note that if $A_{d}\left(1/d,\cdots ,1/d\right)=1/d$, then the corresponding copula must be the comonotonicity copula $M_{d}$. Indeed, if $A_{d}\left(1/d,\cdots ,1/d\right)=1/d$ it follows from (4) that $C_{d}\left(u,\cdots ,u\right)=u$ for every u∈(0,1). Because, for any copula $C_{d}$, it holds that $C_{d}\left(u\right)\leq M_{d}\left(u\right)$ for all $u\in {\left[0,1\right]}^{d}$, the upper Fréchet bound, and $C_{d}\left(u\right)\geq C_{d}\left(\min\left(u_{1},\ldots ,u_{d}\right),\ldots ,\min\left(u_{1},\cdots ,u_{d}\right)\right)$, where the latter quantity equals $\min(u_{1},\cdots ,u_{d})$ in this case and, consequently, $C_{d}\left(u\right)\geq M_{d}\left(u\right)$ for all $u\in {\left[0,1\right]}^{d}$. Hence, in this case $C_{d}=M_{d}$.

Similarly, if $A_{d}\left(1/d,\cdots ,1/d\right)=1$, then the corresponding copula $C_{d}$ must be the independence copula $\Pi_{d}$. To see this, first suppose that there exists a point $w={\left(w_{1},\cdots ,w_{d-1},1-\sum_{j=1}^{d-1}w_{j}\right)}^{⊤}\in \Delta_{d-1}$, such that $A_{d}\left(w\right)=c<1$. Now, define a point $z\in \Delta_{d-1}$ by setting $z_{j}=\left(1-w_{j}\right)/\left(d-1\right)$ for j=1,⋯,d−1 and $z_{d}=1-\sum_{j=1}^{d-1}z_{j}=\sum_{j=1}^{d-1}w_{j}/\left(d-1\right)$. Because $A_{d}$ is a convex function, then

$$\begin{array}{c} 1=A_{d}\left(\frac{1}{d},\cdots ,\frac{1}{d}\right)=A_{d}\left(\frac{1}{d}w+\left(1-\frac{1}{d}\right)z\right)\leq \frac{1}{d}A_{d}\left(w\right)+\frac{d-1}{d}A_{d}\left(z\right)\leq \frac{c+d-1}{d}<1 \end{array}$$

which is a contradiction. This means that $A_{d}\left(w\right)=1$ for every $w\in \Delta_{d-1}$. Immediately from (4), we get that $C_{d}\left(u\right)=\prod_{j=1}^{d}u_{j}$ for every $u\in {\left[0,1\right]}^{d}$ and, hence, $C_{d}=\Pi_{d}$.

Finally, from Definition 1, it follows that the marginal copula of an extreme-value copula is also an extreme-value copula.

We next provide an illustrative example.

**Example** **1.** *Let $C_{d}$ be the d-variate extreme-value copula of ${\left(X_{1},\cdots ,X_{d}\right)}^{⊤}$ and $C_{d+1}$ be the (d+1)-variate copula of ${\left(X_{1},\cdots ,X_{d},X_{d+1}\right)}^{⊤}$ where $X_{d+1}$ is independent of ${\left(X_{1},\cdots ,X_{d}\right)}^{⊤}$, that is*

$$\begin{array}{c} C_{d+1}\left(u_{1},\cdots ,u_{d},u_{d+1}\right)=C_{d}\left(u_{1},\cdots ,u_{d}\right)u_{d+1}. \end{array}$$

*Subsequently, from Definition 1, $C_{d+1}$ is also an extreme-value copula. The stable dependence function $\ell_{d+1}$ can be expressed, using* (3)*, as*

$$\begin{array}{c} \ell_{d+1}\left(x_{1},\cdots ,x_{d+1}\right)=-\log\left(C_{d+1}\left(e^{-x_{1}},\cdots ,e^{-x_{d+1}}\right)\right)=\ell_{d}\left(x_{1},\cdots ,x_{d}\right)+x_{d+1}. \end{array}$$

*Then from* (5)

$$\begin{array}{c} A_{d+1}\left(\frac{x_{1}}{\sum_{j=1}^{d+1}x_{j}},\cdots ,\frac{x_{d+1}}{\sum_{j=1}^{d+1}x_{j}}\right)=\frac{\left(\sum_{j=1}^{d}x_{j}\right)A_{d}\left(\frac{x_{1}}{\sum_{j=1}^{d}x_{j}},\cdots ,\frac{x_{d}}{\sum_{j=1}^{d}x_{j}}\right)+x_{d+1}}{\sum_{j=1}^{d+1}x_{j}} \end{array}$$

*and in particular*

$$\begin{array}{c} A_{d+1}\left(\frac{1}{d+1},\cdots ,\frac{1}{d+1}\right)=\frac{1}{d+1}\left(dA_{d}\left(\frac{1}{d},\cdots ,\frac{1}{d}\right)+1\right). \end{array}$$

Another class of copulas that we consider is the class of multivariate Archimedean copulas, thoroughly discussed, for example, in [12].

**Definition** **2** (Archimedean copula)**.** *A non-increasing and continuous function ψ:[0,∞)→[0,1], which satisfies the conditions ψ(0)=1, $\lim_{x\to \infty }\psi \left(x\right)=0$ and is strictly decreasing on [0,inf{x:ψ(x)=0}) is called an Archimedean generator. A d-dimensional copula $C_{d}$ is called Archimedean if it, for any $u\in {\left[0,1\right]}^{d}$, permits the representation*

$$\begin{array}{c} C_{d}\left(u\right)=\psi \left[\psi^{-1}\left(u_{1}\right)+\cdots +\psi^{-1}\left(u_{d}\right)\right] \end{array}$$

*for some Archimedean generator ψ and its inverse $\psi^{-1}:\left(0,1\right]\to \left[0,\infty \right)$, where, by convention, ψ(∞)=0 and $\psi^{-1}\left(0\right)=\inf\left\{u:\psi (u)=0\right\}$.*

In [12], the authors also provide a characterization of an Archimedean generator leading to some Archimedean copula by means of the following definition and proposition.

**Definition** **3** (*d*-monotone function)**.** *A real function f is called d-monotone on the interval [0,∞), where d≥2, if it is continuous on [0,∞) and differentiable on (0,∞) up to the order d−2 and the derivatives satisfy*

$$\begin{array}{c} {\left(-1\right)}^{k}f^{\left(k\right)}\left(x\right)\geq 0,\;\;\;\mathrm{for}\;k=0,1,\cdots d-2 \end{array}$$

*for any x∈(0,∞) and further if ${\left(-1\right)}^{d-2}f^{\left(d-2\right)}$ is non-increasing and convex in (0,∞). If f has derivatives of all orders in (0,∞) and if ${\left(-1\right)}^{k}f^{\left(k\right)}\left(x\right)\geq 0$ for any x∈(0,∞) and any k=0,1,⋯, then f is called completely monotone.*

It can be shown that exactly this definition is the key to specify which Archimedean generators can generate copulas.

**Proposition** **1** (Characterization of Archimedean copulas)**.** *Let ψ be an Archimedean generator and d≥2. Subsequently, $C_{d}:{\left[0,1\right]}^{d}\to \left[0,1\right]$ given by*

$$\begin{array}{c} C_{d}\left(u\right)=\psi \left[\psi^{-1}\left(u_{1}\right)+\cdots +\psi^{-1}\left(u_{d}\right)\right] \end{array}$$

*is a d-dimensional copula if and only if ψ is d-monotone on [0,∞).*

**Corollary** **1.** *An Archimedean generator ψ can generate a copula in any dimension if and only if it is completely monotone.*

Most of the well-known Archimedean generators are completely monotone, also called strict generators. For strict generators, $\psi^{-1}\left(0\right)=\infty$. However, the range of parameter values possibly depends on the dimension. We illustrate this with the Clayton copula family.

**Example** **2.** *Let $C_{d}$ be the d-variate Clayton copula with parameter θ. In the bivariate case, its generator is defined as $\psi_{\theta }\left(t\right)={\left(1+\theta t\right)}_{+}^{-1/\theta }$ with θ≥−1. However, $\psi_{\theta }$ is d-monotone only for θ≥−1/(d−1) (see [12]). That is, if we want to consider Clayton copula in any dimension, we have to restrict ourselves to θ≥0, where case θ=0 is defined as a limit θ↘0 and, in fact, corresponds to the independence copula.*

**Figure 1:**
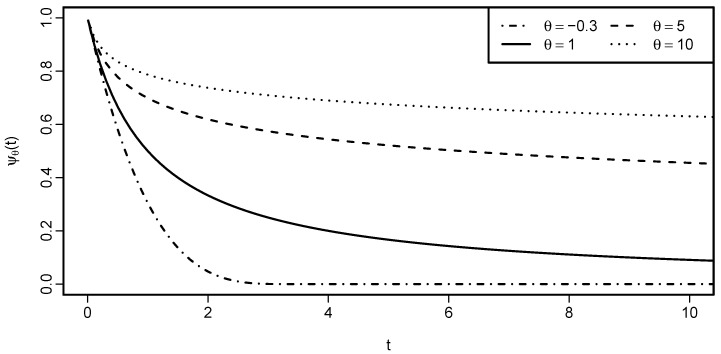
Generator of Clayton copula with parameters −0.3 (dash-dotted line), 1 (solid line), 5 (dashed line) and 10 (dotted line).

*Figure 1 shows how the generator of the Clayton family depends on the parameter θ. When θ<0 and, thus, $\psi_{\theta }$ is not completely monotone, then there exists t∈(0,∞), such that $\psi_{\theta }\left(t\right)=0$. Otherwise, for θ≥0, $\lim_{t\to \infty }\psi_{\theta }\left(t\right)=0$, but for every t∈(0,∞) we have $\psi_{\theta }\left(t\right)>0$.*

In Figure 1, we see the most common shape of the generator function. The following lemma focuses on the behavior of generators close to t=0 and is useful later in this text.

**Lemma** **1.** *Let ψ be an Archimedean generator that generates a copula, differentiable on (0,ϵ) for some ϵ>0. Afterwards, $\psi^{\prime }\left(0^{+}\right)=\lim_{t↘0}\psi^{\prime }\left(t\right)$ can take values in [−∞,0).*

**Proof.**  It can be easily shown that ψ is a convex function on [0,∞) [13] (Theorem 6.3.3). That means that $\psi^{\prime }$ is a non-decreasing function on [0,∞). Additionally, from Definition 2, ψ is strictly decreasing on [0,inf{x:ψ(x)=0}). That is, $\psi^{\prime }$ is negative on (0,inf{x:ψ(x)=0}), which implies that $\psi^{\prime }\left(0^{+}\right)\leq 0$. Suppose now that $\psi^{\prime }\left(0^{+}\right)=0$. Afterwards, from negativity of $\psi^{\prime }$ on (0,inf{x:ψ(x)=0}), $\psi^{\prime }$ must decrease, which is in contradiction with the fact that $\psi^{\prime }$ is a non-decreasing function on [0,∞). □

The following example shows that $\psi^{\prime }\left(0^{+}\right)$ can be equal to −∞.

**Example** **3.** *Let $\psi_{\theta }\left(t\right)=\exp\left(-t^{1/\theta }\right)$ for θ≥1 which is the generator of the Gumbel-Hougaard family. Then*

ψθ′(0+)=limt↘0−1θexp(−t1/θ)t1/θ−1=−1,∞ifθ=1,−∞,1ifθ>1.

**Figure 2:**
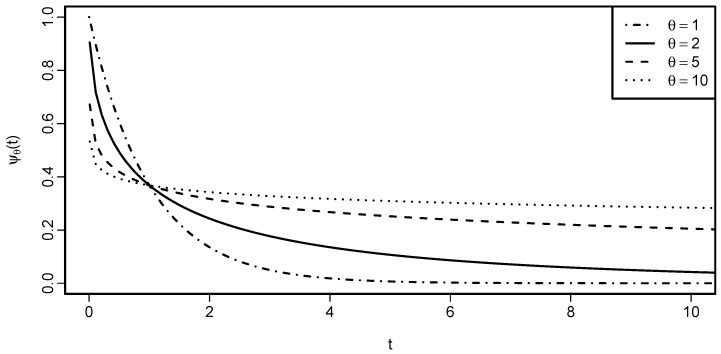
Generator of the Gumbel-Hougaard copula with parameters 1 (dash-dotted line), 2 (solid line), 5 (dashed line) and 10 (dotted line).

*Recall that θ=1 corresponds to the independence copula. Figure 2 shows how the generator of Gumbel-Hougaard family depends on the parameter θ.*

## 3. Tail Coefficients

In the bivariate case (i.e., d=2), lower and upper tail coefficients are defined, respectively, as

$$\begin{array}{cc} \lambda_{L}\left(C_{2}\right) & =\lim_{u↘0}P\left(U_{2}\leq u|U_{1}\leq u\right)=\lim_{u↘0}P\left(U_{1}\leq u|U_{2}\leq u\right)=\lim_{u↘0}\frac{C_{2}\left(u,u\right)}{u},\\ \lambda_{U}\left(C_{2}\right) & =\lim_{u↗1}P\left(U_{2}>u|U_{1}>u\right)=\lim_{u↗1}P\left(U_{1}>u|U_{2}>u\right)=\lim_{u↗1}\frac{1-2u+C_{2}\left(u,u\right)}{1-u}, \end{array}$$

if the limits above exist. Throughout the text, when defining these and other tail coefficients, we will assume the existence of the limits involved. The general idea behind the tail coefficients is to measure how likely a random variable is extreme, given that another variable is extreme. These coefficients can take values between 0 and 1, since they are probabilities.

For extreme-value copulas, tail coefficients can be expressed as functions of Pickands dependence function $A_{2}$ corresponding to the copula $C_{2}$ as

(6) $$\begin{array}{c} \begin{array}{cc} \lambda_{L}\left(C_{2}\right) & =\left\{\begin{array}{cc} 1 & \mathrm{if}A_{2}\left(1/2,1/2\right)=1/2,\\ 0 & \mathrm{otherwise}, \end{array}\right.\\ \lambda_{U}\left(C_{2}\right) & =2(1-A_{2}\left(1/2,1/2\right)), \end{array} \end{array}$$

see [11]. That is, unless the studied copula is the comonotonicity copula, extreme-value copulas do not possess any lower tail dependence. Recall that, when $A_{2}\left(1/2,1/2\right)=1$, the corresponding copula must be the independence copula $\Pi_{2}$. Therefore, an extreme-value copula possesses upper tail dependence, unless the copula is the independence copula.

In case of Archimedean copulas, the tail coefficients can be expressed via the corresponding generator ψ as

$$\begin{array}{cc} \lambda_{L}\left(C_{2}\right) & =2\lim_{u↘0}\frac{\psi^{\prime }\left(2\psi^{-1}\left(u\right)\right)}{\psi^{\prime }\left(\psi^{-1}\left(u\right)\right)},\\ \lambda_{U}\left(C_{2}\right) & =2-2\lim_{u↗1}\frac{\psi^{\prime }\left(2\psi^{-1}\left(u\right)\right)}{\psi^{\prime }\left(\psi^{-1}\left(u\right)\right)}=2-2\lim_{t↘0}\frac{\psi^{\prime }\left(2t\right)}{\psi^{\prime }\left(t\right)}, \end{array}$$

see [14]. Note that both tail coefficients only depend on the behavior of the generator ψ in proximity of the points 0 and $\psi^{-1}\left(0\right)$. Recall that, in the case of strict Archimedean generators, the latter is equal to *∞*.

Given their meaning and mathematical expression, tail coefficients cannot be generalized in general dimension d≥2 in a straightforward and unique way. We first propose a set of desirable properties that are expected to hold for any multivariate tail coefficient ${𝓉}_{d}:\mathrm{Cop}\left(d\right)\to R$ and for any *d*-variate copulas $C_{d}$ and $C_{d,m}$, m=1,2,⋯. The following properties are stated under the working condition that all tail coefficients (${𝓉}_{d}\left(C_{d}\right)$, ${𝓉}_{d+1}\left(C_{d+1}\right)$, ${𝓉}_{d}\left(C_{d,m}\right)$, and so on) exist.

(*T*_1_) (Normalization) ${𝓉}_{d}\left(M_{d}\right)=1,{𝓉}_{d}\left(\Pi_{d}\right)=0$,
(*T*_2_) (Continuity) If $\lim_{m\to \infty }C_{d,m}\left(u\right)=C_{d}\left(u\right),\forall u\in {\left[0,1\right]}^{d}$, then ${𝓉}_{d}\left(C_{d,m}\right)\to {𝓉}_{d}\left(C_{d}\right)$ as m→∞,
(*T*_3_) (Permutation invariance) ${𝓉}_{d}\left(C_{d}^{\pi }\right)={𝓉}_{d}\left(C_{d}\right)$ for every permutation π,
(*T*_4_) (Addition of an independent component) For $X_{d+1}$ independent of $\left(X_{1},\cdots ,X_{d}\right)$
  $$\begin{array}{c} {𝓉}_{d}\left(C_{d}\right)\geq {𝓉}_{d+1}\left(C_{d+1}\right). \end{array}$$
  Property $\left(T_{4}\right)$ could be formulated in a slightly stricter way, as
$\left(T_{4}^{\prime }\right)$ For $X_{d+1}$, independent of $\left(X_{1},\cdots ,X_{d}\right)$, there exists a constant $k_{d}\left({𝓉}_{d}\right)\in \left[0,1\right]$ not depending on $C_{d}$ such that
  $$\begin{array}{c} {𝓉}_{d+1}\left(C_{d+1}\right)=k_{d}\left({𝓉}_{d}\right)\cdot {𝓉}_{d}\left(C_{d}\right). \end{array}$$
  Because both lower and upper tail dependence are of interest, usually we consider that ${𝓉}_{d}$ has actually two versions ${𝓉}_{U,d}$ and ${𝓉}_{L,d}$ focusing on either upper tail (variables simultaneously large) or lower tail (variables simultaneously small) dependence respectively. Thus we can also consider the following property
(*T*_5_) (Duality) ${𝓉}_{L,d}\left(C_{d}^{S}\right)={𝓉}_{U,d}\left(C_{d}\right)$.

In general, some of the desirable properties above are easy to be enforced. If one starts with a candidate coefficient ${𝓉}_{d}^{*}$, property $\left(T_{1}\right)$ can be achieved by defining

$$\begin{array}{c} {𝓉}_{d}\left(C_{d}\right)=\frac{{𝓉}_{d}^{*}\left(C_{d}\right)-{𝓉}_{d}^{*}\left(\Pi_{d}\right)}{{𝓉}_{d}^{*}\left(M_{d}\right)-{𝓉}_{d}^{*}\left(\Pi_{d}\right)}. \end{array}$$

Property $\left(T_{3}\right)$ can be achieved by taking an average of the candidate coefficient ${𝓉}_{d}^{*}$ over all of the permutations

$$\begin{array}{c} {𝓉}_{d}\left(C_{d}\right)=\frac{1}{d!}\sum_{\pi \in S_{d}}{𝓉}_{d}^{*}\left(C_{d}^{\pi }\right), \end{array}$$

where $S_{d}$ denotes all of the permutations of the set {1,⋯,d}. Note, however, that, especially for high dimensions, this significantly increases computational complexity. In the case of property $\left(T_{5}\right)$, we can simply use it to define an upper tail coefficient from the lower tail one (or the other way around).

In the following, we briefly review multivariate tail coefficients proposed in the literature and elaborate on their behavior with respect to the desirable properties $\left(T_{1}\right)$–$\left(T_{5}\right)$. For brevity of presentation, we refer to $\left(T_{4}\right)$ or its variant $\left(T_{4}^{\prime }\right)$ as the “addition property”. To simplify the notation, the subscript *d* of ${𝓉}_{d}$, denoting the dimension, will sometimes be omitted in the text, the dimension being clear from an argument of a functional 𝓉.

### 3.1. Frahm’s Extremal Dependence Coefficient

Frahm (see [3]) considered lower and upper extremal dependence coefficients $\epsilon_{L},\epsilon_{U}$, respectively, defined as

(7) $$\begin{array}{c} \begin{array}{cc} \epsilon_{L}\left(C_{d}\right) & =\lim_{u↘0}P\left(U_{\max}\leq u|U_{\min}\leq u\right)=\lim_{u↘0}\frac{P(U_{\max}\leq u)}{P(U_{\min}\leq u)}=\lim_{u↘0}\frac{C_{d}\left(u1\right)}{1-{\bar{C}}_{d}\left(u1\right)},\\ \epsilon_{U}\left(C_{d}\right) & =\lim_{u↗1}P\left(U_{\min}>u|U_{\max}>u\right)=\lim_{u↗1}\frac{P(U_{\min}>u)}{P(U_{\max}>u)}=\lim_{u↗1}\frac{{\bar{C}}_{d}\left(u1\right)}{1-C_{d}\left(u1\right)}, \end{array} \end{array}$$

given the limits exist, where $U_{\max}=\max\left(U_{1},\cdots ,U_{d}\right)$ and $U_{\min}=\min\left(U_{1},\cdots ,U_{d}\right)$. These coefficients are not equal to $\lambda_{L},\lambda_{U}$, respectively, in the bivariate case. More specifically, for any copula $C_{2}$ (see [3])

$$\begin{array}{c} \epsilon_{L}\left(C_{2}\right)=\frac{\lambda_{L}\left(C_{2}\right)}{2-\lambda_{L}\left(C_{2}\right)},\;\;\epsilon_{U}\left(C_{2}\right)=\frac{\lambda_{U}\left(C_{2}\right)}{2-\lambda_{U}\left(C_{2}\right)}. \end{array}$$

Thus, we can consider it more as a different type of tail dependence coefficient than a generalization of bivariate tail coefficients.

For extreme-value copulas, extremal dependence coefficients can be stated in terms of Pickands dependence function. Let $C_{d}$ be an extreme-value copula with Pickands dependence function $A_{d}$ and denote the Pickands dependence function of the marginal copula $C_{j}^{\left(k_{1},\cdots ,k_{j}\right)}$ as $A_{j}^{\left(k_{1},\cdots ,k_{j}\right)}$. Subsequently,

$$\begin{array}{cc} C_{d}\left(t,\cdots ,t\right) & =\exp\left\{d\log\left(t\right)A_{d}\left(1/d,\cdots ,1/d\right)\right\}=t^{dA_{d}\left(1/d,\cdots ,1/d\right)} \end{array}$$

(8) $$\begin{array}{cc} {\bar{C}}_{d}\left(t,\cdots ,t\right) & =1+\sum_{j=1}^{d}{\left(-1\right)}^{j}\sum_{1\leq k_{1}<\cdots <k_{j}\leq d}t^{jA_{j}^{\left(k_{1},\cdots ,k_{j}\right)}\left(1/j,\cdots ,1/j\right)} \end{array}$$

(9) $$\begin{array}{cc} \; & =1+\sum_{j=1}^{d}{\left(-1\right)}^{j}\sum_{1\leq k_{1}<\cdots <k_{j}\leq d}t^{jA_{d}\left(w_{1},\cdots ,w_{d}\right)}, \end{array}$$

where $w_{\ell }=1/j$ if $\ell \in \{k_{1},\cdots ,k_{j}\}$ and $w_{\ell }=0$ otherwise. As opposed to (8), expression (9) only involves the overall *d*-dimensional Pickands dependence function. This might be helpful, for example, during estimation, since not all of the lower-dimensional Pickands dependence functions in (8) need to be estimated.

Thus, for the lower extremal dependence coefficient, one obtains

(10) $$\begin{array}{cc} \epsilon_{L}\left(C_{d}\right) & =\lim_{t↘0}\frac{t^{dA_{d}\left(1/d,\cdots ,1/d\right)}}{-\sum_{j=1}^{d}{\left(-1\right)}^{j}\sum_{1\leq k_{1}<\cdots <k_{j}\leq d}t^{jA_{j}^{\left(k_{1},\cdots ,k_{j}\right)}\left(1/j,\cdots ,1/j\right)}}=\left\{\begin{array}{cc} 1 & if\;A_{d}\left(1/d,\cdots ,1/d\right)=1/d,\\ 0 & otherwise \end{array}\right. \end{array}$$

because the polynomial (in *t*) in the denominator contains lower-degree terms than the polynomial in the numerator. We can see that this behavior resembles $\lambda_{L}$ for bivariate extreme-value copulas, since the only extreme-value copula possessing lower tail dependence is the comonotonicity copula.

For the upper extremal dependence coefficient, we can calculate

(11) $$\begin{array}{ll} \epsilon_{U}\left(C_{d}\right) & =\lim_{t↗1}\frac{1+\sum_{j=1}^{d}{\left(-1\right)}^{j}\sum_{1\leq k_{1}<\cdots <k_{j}\leq d}t^{jA_{j}^{\left(k_{1},\cdots ,k_{j}\right)}\left(1/j,\cdots ,1/j\right)}}{1-t^{dA_{d}\left(1/d,\cdots ,1/d\right)}}\\ \; & =\lim_{t↗1}\frac{\sum_{j=1}^{d}{\left(-1\right)}^{j}\sum_{1\leq k_{1}<\cdots <k_{j}\leq d}jA_{j}^{\left(k_{1},\cdots ,k_{j}\right)}\left(1/j,\cdots ,1/j\right)t^{jA_{j}^{\left(k_{1},\cdots ,k_{j}\right)}\left(1/j,\cdots ,1/j\right)-1}}{-dA_{d}\left(1/d,\cdots ,1/d\right)t^{dA_{d}\left(1/d,\cdots ,1/d\right)-1}}\\ \; & =\frac{\sum_{j=1}^{d}{\left(-1\right)}^{j+1}\sum_{1\leq k_{1}<\cdots <k_{j}\leq d}jA_{j}^{\left(k_{1},\cdots ,k_{j}\right)}\left(1/j,\cdots ,1/j\right)}{dA_{d}\left(1/d,\cdots ,1/d\right)}\\ \; & =\frac{\sum_{j=1}^{d}{\left(-1\right)}^{j+1}\sum_{1\leq k_{1}<\cdots <k_{j}\leq d}jA_{d}\left(w_{1},\cdots ,w_{d}\right)}{dA_{d}\left(1/d,\cdots ,1/d\right)}, \end{array}$$

where, as above, $w_{\ell }=1/j$ if $\ell \in \{k_{1},\cdots ,k_{j}\}$ and $w_{\ell }=0$ otherwise.

We next look into the tail coefficients (7) for Archimedean copulas. Let ${\left\{C_{d}\right\}}_{d\geq 2}$ be a sequence of *d*-dimensional Archimedean copulas with (the same) generator ψ. Subsequently,

Cd(u,⋯,u)=ψ(dψ−1(u)),C¯d(u,⋯,u)=1+∑j=1d(−1)jdjψ(jψ−1(u)).

The corresponding derivatives, if they exist, are

Cd′(u,⋯,u)=ψ′(dψ−1(u))d(ψ−1)′(u),C¯d′(u,⋯,u)=∑j=1d(−1)jdjψ′(jψ−1(u))j(ψ−1)′(u).

Afterwards, the extremal dependence coefficients can be expressed as

(12) ϵL(Cd)=limu↘0Cd(u1)1−C¯d(u1)=limu↘0ψ(dψ−1(u))∑j=1d(−1)j+1djψ(jψ−1(u))=limu↘0ψ′(dψ−1(u))d∑j=1d(−1)j+1djψ′(jψ−1(u))j,

(13) ϵU(Cd)=limu↗1C¯d(u1)1−Cd(u1)=limu↗11+∑j=1d(−1)jdjψ(jψ−1(u))1−ψ(dψ−1(u))=limu↗1∑j=1d(−1)jdjψ′(jψ−1(u))j−ψ′(dψ−1(u))d=limt↘0∑j=1d(−1)jdjψ′(jt)j−ψ′(dt)d,

where we used L’Hospital’s rule to get to the equation in (12), and the second equation in the derivation towards (13). Recall that $\psi^{-1}\left(1\right)=0$ and $\psi^{-1}\left(0\right)=\inf\left\{u:\psi \left(u\right)=0\right\}$. One can see that using L’Hospital’s rule does not solve the 0/0 limit problem for general ψ and knowledge of the precise behavior of ψ is thus crucial for calculating the coefficients $\epsilon_{L}\left(C_{d}\right)$ and $\epsilon_{U}\left(C_{d}\right)$.

As will be illustrated in Section 6, Archimedean copulas can have both extremal dependence coefficients non-zero, depending on the generator. For $\epsilon_{U}$, one additional assumption regarding a generator ψ may become useful. Because (from the definition of the generator) $\lim_{u↗1}\psi^{-1}\left(u\right)=0$, if the additional condition $\psi^{\prime }\left(0^{+}\right)>-\infty$ is fulfilled, we get

ϵU(Cd)=∑j=1d(−1)jdjψ′(0+)j−ψ′(0+)d=∑j=1d(−1)jd−1j−1=0,

using that from Lemma 1 $\psi^{\prime }\left(0^{+}\right)$ cannot be equal to zero. In other words, if $\psi^{\prime }\left(0^{+}\right)>-\infty$, then the corresponding Archimedean copula is upper tail independent, for every dimension.

Next, we investigate which of the desirable properties $\left(T_{1}\right)$–$\left(T_{5}\right)$ are satisfied for Frahm’s extremal dependence coefficients $\epsilon_{L}$ and $\epsilon_{U}$.

**Proposition** **2.** *Frahm’s extremal dependence coefficients $\epsilon_{L}$ and $\epsilon_{U}$ satisfy normalization property $\left(T_{1}\right)$, permutation invariance property $\left(T_{3}\right)$, and addition property $\left(T_{4}^{\prime }\right)$, with $k_{d}\left(\epsilon_{L}\right)=k_{d}\left(\epsilon_{U}\right)=0$ for every d≥2, and $\left(T_{5}\right)$.*

**Proof.**  Normalization property $\left(T_{1}\right)$ follows from straightforward calculations

$$\begin{array}{c} \begin{array}{cc} \epsilon_{L}\left(M_{d}\right) & =\lim_{u↘0}\frac{u}{1-(1-u)}=1,\\ \epsilon_{L}\left(\Pi_{d}\right) & =\lim_{u↘0}\frac{u^{d}}{1-{\left(1-u\right)}^{d}}=0, \end{array}\begin{array}{cc} \epsilon_{U}\left(M_{d}\right) & =\lim_{u↗1}\frac{1-u}{1-u}=1,\\ \epsilon_{U}\left(\Pi_{d}\right) & =\lim_{u↗1}\frac{{\left(1-u\right)}^{d}}{1-u^{d}}=0. \end{array} \end{array}$$

Permutation invariance property $\left(T_{3}\right)$ follows immediately from the fact that the coefficients only depend on $U_{\max}$ and $U_{\min}$, which do not depend on the order of components of the random vector.

Look now into the addition of an independent component, i.e., property $\left(T_{4}^{\prime }\right)$. To be able to distinguish between the dimensions, we use the notation $U_{\max,d}=\max\left(U_{1},\cdots ,U_{d}\right)$ and $U_{\min,d}=\min\left(U_{1},\cdots ,U_{d}\right)$. For $X_{d+1}$ independent of $\left(X_{1},\cdots ,X_{d}\right)$, we have $P\left(U_{\min,d+1}\leq u\right)\geq P\left(U_{\min,d}\leq u\right)$ and $P\left(U_{\max,d+1}>u\right)\geq P\left(U_{\max,d}>u\right)$ for every u∈[0,1]. Further, $P\left(U_{\max,d+1}\leq u\right)=P\left(U_{\max,d}\leq u,U_{d+1}\leq u\right)=uP\left(U_{\max,d}\leq u\right)$ and similarly $P\left(U_{\min,d+1}>u\right)=P\left(U_{\min,d}>u,U_{d+1}>u\right)=\left(1-u\right)P\left(U_{\min,d}>u\right)$. Thus,

$$\begin{array}{cc} \epsilon_{L}\left(C_{d+1}\right) & =\lim_{u↘0}\frac{P(U_{\max,d+1}\leq u)}{P(U_{\min,d+1}\leq u)}\leq \lim_{u↘0}\frac{uP(U_{\max,d}\leq u)}{P(U_{\min,d}\leq u)}=0\cdot \epsilon_{L}\left(C_{d}\right)=0,\\ \epsilon_{U}\left(C_{d+1}\right) & =\lim_{u↗1}\frac{P(U_{\min,d+1}>u)}{P(U_{\max,d+1}>u)}\leq \lim_{u↗1}\frac{\left(1-u\right)P\left(U_{\min,d}>u\right)}{P(U_{\max,d}>u)}=0\cdot \epsilon_{U}\left(C_{d}\right)=0, \end{array}$$

which means that the property about adding an independent component $\left(T_{4}^{\prime }\right)$ holds with constants $k_{d}\left(\epsilon_{L}\right)=k_{d}\left(\epsilon_{U}\right)=0$ for every d≥2.

We next look into duality $\left(T_{5}\right)$. Using relation (1) between the survival function and the survival copula, coefficients $\epsilon_{L}$ and $\epsilon_{U}$ can be rewritten as

$$\begin{array}{cc} \epsilon_{L}\left(C_{d}\right) & =\lim_{u↘0}\frac{C(u1)}{1-\bar{C}\left(u1\right)}=\lim_{u↘0}\frac{C(u1)}{1-C^{S}\left(1-u1\right)},\\ \epsilon_{U}\left(C_{d}\right) & =\lim_{u↗1}\frac{\bar{C}\left(u1\right)}{1-C(u1)}=\lim_{u↗1}\frac{C^{S}\left(1-u1\right)}{1-C(u1)} \end{array}$$

and thus

$$\begin{array}{cc} \epsilon_{L}\left(C_{d}^{S}\right) & =\lim_{u↘0}\frac{C^{S}\left(u1\right)}{1-C(1-u1)}=\lim_{v↗1}\frac{C^{S}\left(1-v1\right)}{1-C(v1)}=\epsilon_{U}\left(C_{d}\right), \end{array}$$

where substitution v=1−u was used. This proves the validity of duality property $\left(T_{5}\right)$. □

We suspect that the continuity property $\left(T_{2}\right)$ does not hold in its full generality for most multivariate tail coefficients. To obtain insight into this, consider the following example with a sequence of copulas $\left\{C_{d,m}\right\}$ given by

$$C_{d,m}\left(u\right)=M_{d}\left(u\right)𝟙\left\{\min\left\{u_{1},\cdots ,u_{d}\right\}\leq \frac{1}{m}\right\}+\left(\frac{1}{m}+\frac{\Pi_{d}\left(u-\frac{1}{m}1\right)}{{\left(1-\frac{1}{m}\right)}^{d-1}}\right)𝟙\left\{\min\left\{u_{1},\cdots ,u_{d}\right\}>\frac{1}{m}\right\}.$$

Note that the distribution that is given by $C_{d,m}$ is uniform on the set ${\left[\frac{1}{m},1\right]}^{d}$ and it corresponds to the upper Fréchet’s bound $M_{d}$ otherwise. Note that $C_{d,m}$ is a copula with an ordinal sum representation, see [8] (Section 3.2.2).

It is easily seen that $C_{d,m}\to \Pi_{d}$ as m→∞ uniformly on ${\left[0,1\right]}^{d}$. Note that $\epsilon_{L}\left(C_{d,m}\right)=1$ for each m∈N. On the other hand, $\epsilon_{L}\left(\Pi_{d}\right)=0$. Hence, for this sequence of copulas, the continuity property $\left(T_{2}\right)$ does not hold.

However, a continuity property may hold, in general, under more specific conditions on the copula sequences. One such condition is that of a sequence of contaminated copulas, defined as follows.

Let $C_{d}$ and $B_{d,m}$, for m=1,⋯ be *d*-variate copulas, and let $\epsilon_{m}$ be a sequence of numbers in [0,1]. One considers the sequence of contaminated copulas

(14) $$C_{d,m}=\left(1-\epsilon_{m}\right)C_{d}+\epsilon_{m}B_{d,m}.$$

Note that $C_{d,m}$ is a convex combination of the copulas $C_{d}$ and $B_{d,m}$ and, hence, is also a copula, see e.g., [8]. The interest is to investigate the behavior of a tail coefficient for the sequence $C_{d,m}$ when $\epsilon_{m}\to 0$, as m→∞.

Proposition 3 establishes a continuity property for Frahm’s extremal dependence coefficient.

**Proposition** **3.** *Suppose that, for any d-variate copulas $C_{d}$ and $C_{d,m}$, m=1,2,⋯, there exist ϵ>0, such that*

(15) $$\begin{array}{c} \frac{C_{d,m}\left(u1\right)}{1-{\bar{C}}_{d,m}\left(u1\right)}\to \frac{C_{d}\left(u1\right)}{1-{\bar{C}}_{d}\left(u1\right)}\;\mathrm{uniformly}\;\mathrm{on}\;\left(0,\epsilon \right),\;\mathrm{as}\;m\to \infty . \end{array}$$

*Further assume that $\epsilon_{L}\left(C_{d,m}\right)$ exists for every m=1,2,⋯. Subsequently, $\epsilon_{L}\left(C_{d,m}\right)\to \epsilon_{L}\left(C_{d}\right)$ as m→∞.*

*In particular, condition* (15) *is satisfied for a sequence of contaminated copulas, as in* (14)*, for which $\epsilon_{m}\to 0$, as m→∞, and provided $\epsilon_{L}\left(C_{d}\right)$ exists.*

**Proof.**  Assumption (15) allows for us to use the Moore–Osgood theorem to interchange the limits and, thus

$$\begin{array}{c} \lim_{m\to \infty }\epsilon_{L}\left(C_{d,m}\right)=\lim_{m\to \infty }\lim_{u↘0}\frac{C_{d,m}\left(u1\right)}{1-{\bar{C}}_{d,m}\left(u1\right)}=\lim_{u↘0}\lim_{m\to \infty }\frac{C_{d,m}\left(u1\right)}{1-{\bar{C}}_{d,m}\left(u1\right)}=\epsilon_{L}\left(C_{d}\right). \end{array}$$

Suppose now that we have a sequence of contaminated copulas, for which $\epsilon_{m}\to 0$, as m→∞. Subsequently, one calculates

(16) $$\begin{array}{cc} \frac{C_{d,m}\left(u1\right)}{1-{\bar{C}}_{d,m}\left(u1\right)}-\frac{C_{d}\left(u1\right)}{1-{\bar{C}}_{d}\left(u1\right)}\; & =\frac{C_{d,m}\left(u1\right)-C_{d}\left(u1\right)}{1-{\bar{C}}_{d,m}\left(u1\right)}+\frac{C_{d}\left(u1\right)}{1-{\bar{C}}_{d,m}\left(u1\right)}-\frac{C_{d}\left(u1\right)}{1-{\bar{C}}_{d}\left(u1\right)}\\ \; & =\frac{\varepsilon_{m}\left(B_{d,m}\left(u1\right)-C_{d}\left(u1\right)\right)}{1-{\bar{C}}_{d,m}\left(u1\right)}+\frac{C_{d}\left(u1\right)\varepsilon_{m}\left({\bar{B}}_{d,m}\left(u1\right)-{\bar{C}}_{d}\left(u1\right)\right)}{\left(1-{\bar{C}}_{d,m}\left(u1\right)\right)\left(1-{\bar{C}}_{d}\left(u1\right)\right)}. \end{array}$$

One next realizes that $\max\{B_{d,m}\left(u1\right),C_{d}\left(u1\right)\}\leq u$ and $\min\{1-{\bar{C}}_{d,m}\left(u1\right),1-{\bar{C}}_{d}\left(u1\right)\}\geq u$. Furthermore, with the help of Formula (2) for the survival function of a copula one gets ${\bar{B}}_{d,m}\left(u1\right)-{\bar{C}}_{d}\left(u1\right)=O\left(u\right)$. Thus, one can bound

$$|\frac{C_{d,m}\left(u1\right)}{1-{\bar{C}}_{d,m}\left(u1\right)}-\frac{C_{d}\left(u1\right)}{1-{\bar{C}}_{d}\left(u1\right)}|\leq \frac{\varepsilon_{m}\;u}{u}+\frac{u\;\varepsilon_{m}\;O\left(u\right)}{u^{2}}=\varepsilon_{m}\;O\left(1\right),$$

which implies (15). □

Analogously, a similar result could be stated for $\epsilon_{U}$.

### 3.2. Li’s Tail Dependence Parameter

Suppose that $\emptyset \neq I_{h}\subset \left\{1,\cdots ,d\right\}$ is a subset of indices, such that $|I_{h}|=h$ and $J_{d-h}=\left\{1,\cdots ,d\right\}\backslash I_{h}$. Subsequently, Li [4] (Def. 1.2) defines so-called lower and upper tail dependence parameters, as follows

$$\begin{array}{cc} \lambda_{L}^{I_{h}|J_{d-h}}\left(C_{d}\right) & =\lim_{u↘0}P\left(U_{i}\leq u,\forall i\in I_{h}|U_{j}\leq u,\forall j\in J_{d-h}\right),\\ \lambda_{U}^{I_{h}|J_{d-h}}\left(C_{d}\right) & =\lim_{u↗1}P\left(U_{i}>u,\forall i\in I_{h}|U_{j}>u,\forall j\in J_{d-h}\right), \end{array}$$

given the expressions exist. It is evident that these coefficients heavily depend on the choice of the set $I_{h}$. Additionally, this generalization includes the usual bivariate tail dependence coefficients $\lambda_{L}$ and $\lambda_{U}$, by letting h=1, $I_{1}=\left\{1\right\}$ and $J_{1}=\left\{2\right\}$ or the other way around. Li [4] further states that $\lambda_{L}^{I_{h}|J_{d-h}}\left(C_{d}\right)=\lambda_{U}^{I_{h}|J_{d-h}}\left(C_{d}^{S}\right)$ and, therefore, duality property $\left(T_{5}\right)$ is fulfilled.

One can also notice that, for exchangeable copulas (i.e., symmetric in their arguments), the dependence parameters are in fact functions of cardinality *h* rather than particular contents of $I_{h}$. Especially in this case, it is worth introducing another notation being

$$\begin{array}{cc} \lambda_{L}^{1,\cdots ,h|h+1,\cdots ,d}\left(C_{d}\right) & =\lim_{u↘0}P\left(U_{1}\leq u,\cdots ,U_{h}\leq u|U_{h+1}\leq u,\cdots U_{d}\leq u\right),\\ \lambda_{U}^{1,\cdots ,h|h+1,\cdots ,d}\left(C_{d}\right) & =\lim_{u↗1}P\left(U_{1}>u,\cdots ,U_{h}>u|U_{h+1}>u,\cdots U_{d}>u\right). \end{array}$$

In paper [15], it is shown that these coefficients can be rewritten while using one-sided derivatives of the diagonal section $\delta_{C_{d}}\left(u\right)=C_{d}\left(u,\cdots ,u\right)$ of the corresponding copula in the following way:

$$\begin{array}{cc} \lambda_{L}^{1,\cdots ,h|h+1,\cdots ,d}\left(C_{d}\right) & =\frac{\delta_{C_{d}}^{\prime }\left(0^{+}\right)}{\delta_{(h+1)\cdots d}^{\prime }\left(0^{+}\right)}\\ \lambda_{U}^{1,\cdots ,h|h+1,\cdots ,d}\left(C_{d}\right) & =\frac{\sum_{j=1}^{d}{\left(-1\right)}^{j+1}\sum_{1\leq k_{1}<\cdots <k_{j}\leq d}\delta_{k_{1}\cdots k_{j}}^{\prime }\left(1^{-}\right)}{\sum_{j=1}^{d-h}{\left(-1\right)}^{j+1}\sum_{h+1\leq k_{1}<\cdots <k_{j}\leq d}\delta_{k_{1}\cdots k_{j}}^{\prime }\left(1^{-}\right)} \end{array}$$

where $\delta_{k_{1}\cdots k_{j}}$ denotes the diagonal section of copula $C_{j}^{\left(k_{1},\cdots ,k_{j}\right)}$.

Additionally, the authors in [15] comment on the connection with Frahm’s extremal dependence coefficients $\epsilon_{L}$ and $\epsilon_{U}$, which can be expressed as

$$\begin{array}{cc} \epsilon_{L}\left(C_{d}\right) & =\frac{\delta_{C_{d}}^{\prime }\left(0^{+}\right)}{\sum_{j=1}^{d}{\left(-1\right)}^{j+1}\sum_{1\leq k_{1}<\cdots <k_{j}\leq d}\delta_{k_{1}\cdots k_{j}}^{\prime }\left(0^{+}\right)}\\ \; & =\frac{\lambda_{L}^{1,\cdots ,(d-1)|d}\left(C_{d}\right)}{\sum_{j=1}^{d}{\left(-1\right)}^{j+1}\sum_{1\leq k_{1}<\cdots <k_{j}\leq d}\lambda_{L}^{1,\cdots ,j-1|j}\left(C_{j}^{\left(k_{1},\cdots ,k_{j}\right)}\right)},\\ \epsilon_{U}\left(C_{d}\right) & =\frac{\lambda_{U}^{1,\cdots ,(d-1)|d}\left(C_{d}\right)}{\delta_{C_{d}}^{\prime }\left(1^{-}\right)} \end{array}$$

if all of the above quantities exist.

De Luca and Rivieccio [6] (Def. 2) also use this way to measure tail dependence, although they consider it as a measure for Archimedean copulas only since we can express the measures while using the generator, as

$$\begin{array}{cc} \lambda_{L}^{1,\cdots ,h|h+1,\cdots ,d} & =\lim_{u↘0}\frac{C_{d}\left(u,\cdots ,u\right)}{C_{d-h}^{\left(h+1,\cdots ,d\right)}\left(u,\cdots ,u\right)}=\lim_{u↘0}\frac{\psi (d\psi^{-1}\left(u\right))}{\psi (\left(d-h\right)\psi^{-1}\left(u\right))} \end{array}$$

(17) =limu↘0dψ′(dψ−1(u))(d−h)ψ′((d−h)ψ−1(u)),λU1,⋯,h|h+1,⋯,d=limu↗1C¯d(u,⋯,u)C¯d−h(h+1,⋯,d)(u,⋯,u)=limu↗11+∑j=1d(−1)jdjψ(jψ−1(u))1+∑j=1d−h(−1)jd−hjψ(jψ−1(u))

(18) =limu↗1∑j=1d(−1)jdjψ′(jψ−1(u))j∑j=1d−h(−1)jd−hjψ′(jψ−1(u))j,

where we applied l’Hospital’s rule for obtaining the equation in (17) and (18). In contrast to the Frahm’s coefficient, here the additional condition that $\psi^{\prime }\left(0^{+}\right)>-\infty$ is not helpful, since it leads to

λU1,⋯,h|h+1,⋯,d=∑j=1d(−1)jdjψ′(0+)j∑j=1d−h(−1)jd−hjψ′(0+)j=∑j=1d(−1)jdjj∑j=1d−h(−1)jd−hjj

and numerator and denominator are both equal to zero here.

**Proposition** **4.** *Li’s tail dependence parameters $\lambda_{L}^{I_{h}|J_{d-h}}$ and $\lambda_{U}^{I_{h}|J_{d-h}}$ satisfy normalization property $\left(T_{1}\right)$, addition property $\left(T_{4}\right)$, and duality property $\left(T_{5}\right)$.*

**Proof.**  Duality property $\left(T_{5}\right)$ was shown in [4]. Normalization property $\left(T_{1}\right)$ follows from straightforward calculations while using (17) and (18)

$$\begin{array}{c} \begin{array}{c} \lambda_{L}^{I_{h}|J_{d-h}}\left(M_{d}\right)=\lim_{u↘0}\frac{u}{u}=1,\;\lambda_{L}^{I_{h}|J_{d-h}}\left(\Pi_{d}\right)=\lim_{u↘0}\frac{u^{d}}{u^{d-h}}=0. \end{array} \end{array}$$

For $\lambda_{U}^{I_{h}|J_{d-h}}$, it follows from duality property $\left(T_{5}\right)$.

We now check property $\left(T_{4}\right)$, the addition of an independent random component. Suppose that the added independent component belongs to the set $I_{h+1}$. Subsequently,

$$\begin{array}{c} \lambda_{L}^{I_{h+1}|J_{d-h}}\left(C_{d+1}\right)=\lim_{u↘0}\frac{C_{d}\left(u1\right)u}{C_{d-h}^{J_{d-h}}\left(u1\right)}=0\cdot \lambda_{L}^{I_{h}|J_{d-h}}\left(C_{d}\right)=0. \end{array}$$

If the added independent component belongs to the set $J_{d-h+1}$, then from the definition of the coefficient

$$\begin{array}{c} \lambda_{L}^{I_{h}|J_{d-h+1}}\left(C_{d+1}\right)=\lim_{u↘0}\frac{C_{d}\left(u1\right)u}{C_{d-h}^{J_{d-h}}\left(u1\right)u}=\lambda_{L}^{I_{h}|J_{d-h}}\left(C_{d}\right). \end{array}$$

Showing the duality property for $\lambda_{U}^{I_{h}|J_{d-h}}$ is analogous. □

The proof of Proposition 4 shows that, in fact, property $\left(T_{4}^{\prime }\right)$ is fulfilled if one distinguishes two cases. If the added independent component belongs to the set $I_{h+1}$, then $\left(T_{4}^{\prime }\right)$ holds with $k_{d}\left(\lambda_{L}\right)=k_{d}\left(\lambda_{U}\right)=0$ for every d≥2. However, if the added independent component belongs to the set $J_{d-h+1}$, then $k_{d}\left(\lambda_{L}\right)=k_{d}\left(\lambda_{U}\right)=1$ for every d≥2.

Permutation invariance $\left(T_{3}\right)$ does not hold in general. However, if one would restrict to only permutations that permute indices within $I_{h}$ and within $J_{d-h}$ and not across these two sets, $\lambda_{L}$ and $\lambda_{U}$ would be invariant with respect to such permutations. Further, we might consider the special case when h=d−1, which is if we condition only on one variable. Subsequently, for any permutation π

(19) $$\begin{array}{c} \lambda_{L}^{I_{d-1}|J_{1}}\left(C_{d}^{\pi }\right)=\lim_{u↘0}\frac{C_{d}^{\pi }\left(u1\right)}{u}=\lim_{u↘0}\frac{C_{d}\left(u1\right)}{u}=\lambda_{L}^{I_{d-1}|J_{1}}\left(C_{d}\right) \end{array}$$

and analogously for $\lambda_{U}$, we have $\lambda_{U}^{I_{d-1}|J_{1}}\left(C_{d}^{\pi }\right)=\lambda_{U}^{I_{d-1}|J_{1}}\left(C_{d}\right)$.

A continuity property can be shown under a specific condition on the copula sequence as is established in Proposition 5.

**Proposition** **5.** *Suppose that, for any d-variate copulas $C_{d}$ and $C_{d,m}$, m=1,2,⋯, there exist ϵ>0, such that*

(20) $$\begin{array}{c} \frac{C_{d,m}\left(u1\right)}{C_{d-h,m}^{J_{d-h}}\left(u1\right)}\to \frac{C_{d}\left(u1\right)}{C_{d-h}^{J_{d-h}}\left(u1\right)}\;\mathrm{uniformly}\;\mathrm{on}\;\left(0,\epsilon \right),\;\mathrm{as}\;m\to \infty . \end{array}$$

*Further assume that $\lambda_{L}^{I_{h}|J_{d-h}}\left(C_{d,m}\right)$ exists for every m=1,2,⋯, as well as $\lambda_{L}^{I_{h}|J_{d-h}}\left(C_{d}\right)$. Subsequently, $\lambda_{L}^{I_{h}|J_{d-h}}\left(C_{d,m}\right)\to \lambda_{L}^{I_{h}|J_{d-h}}\left(C_{d}\right)$ as m→∞.*

*In particular, condition* (20) *holds for a sequence of contaminated copulas, see* (14)*, for which $\epsilon_{m}\to 0$, as m→∞, and*

(21) $$\underset{m\to \infty }{lim sup}\underset{u\in (0,\epsilon )}{\sup}\frac{B_{d-h,m}^{J_{d-h}}\left(u1\right)}{C_{d-h}^{J_{d-h}}\left(u1\right)}<\infty ,$$

*and $\lambda_{L}^{I_{h}|J_{d-h}}\left(C_{d}\right)$ exists.*

**Proof.**  The first part of Proposition 5 is proven along the same lines as the proof of Proposition 3 and hence omitted here.

Consider now a sequence of contaminated copulas satisfying in addition (21). We need to show that (20) holds. To see this, note that, similarly as in (16), one gets

(22) $$\frac{C_{d,m}\left(u1\right)}{C_{d-h,m}^{J_{d-h}}\left(u1\right)}-\frac{C_{d}\left(u1\right)}{C_{d-h}^{J_{d-h}}\left(u1\right)}=\frac{\varepsilon_{m}\left(B_{d,m}\left(u1\right)-C_{d}\left(u1\right)\right)}{C_{d-h,m}^{J_{d-h}}\left(u1\right)}+\frac{C_{d}\left(u1\right)\varepsilon_{m}\left(B_{d-h,m}^{J_{d-h}}\left(u1\right)-C_{d-h}^{J_{d-h}}\left(u1\right)\right)}{C_{d-h,m}^{J_{d-h}}\left(u1\right)C_{d-h}^{J_{d-h}}\left(u1\right)}.$$

Further note that, for all sufficiently large *m* for all u∈(0,ϵ)

(23) $$\frac{C_{d-h}^{J_{d-h}}\left(u1\right)}{C_{d,m}^{J_{d-h}}\left(u1\right)}\leq \frac{C_{d-h}^{J_{d-h}}\left(u1\right)}{\left(1-\varepsilon_{m}\right)C_{d-h}^{J_{d-h}}\left(u1\right)}\leq 2.$$

Combining (21), (22) and (23) now yields that (for all sufficiently large *m*)

$$\begin{array}{cc} |\frac{C_{d,m}\left(u1\right)}{C_{d-h,m}^{J_{d-h}}\left(u1\right)}-\frac{C_{d}\left(u1\right)}{C_{d-h}^{J_{d-h}}\left(u1\right)}|\; & \leq \frac{\varepsilon_{m}B_{d,m}\left(u1\right)}{C_{d-h,m}^{J_{d-h}}\left(u1\right)}+\frac{\varepsilon_{m}\;C_{d}\left(u1\right)}{C_{d-h,m}^{J_{d-h}}\left(u1\right)}+\frac{\varepsilon_{m}\;C_{d}\left(u1\right)B_{d-h,m}^{J_{d-h}}\left(u1\right)}{C_{d-h,m}^{J_{d-h}}\left(u1\right)C_{d-h}^{J_{d-h}}\left(u1\right)}+\frac{\varepsilon_{m}\;C_{d}\left(u1\right)}{C_{d-h,m}^{J_{d-h}}\left(u1\right)}\\ \; & \leq \frac{2\;\varepsilon_{m}B_{d,m}\left(u1\right)}{C_{d-h}^{J_{d-h}}\left(u1\right)}+\frac{2\;\varepsilon_{m}\;C_{d}\left(u1\right)}{C_{d-h}^{J_{d-h}}\left(u1\right)}+\frac{2\;\varepsilon_{m}\;C_{d}\left(u1\right)B_{d-h,m}^{J_{d-h}}\left(u1\right)}{C_{d-h}^{J_{d-h}}\left(u1\right)C_{d-h}^{J_{d-h}}\left(u1\right)}+\frac{2\;\varepsilon_{m}\;C_{d}\left(u1\right)}{C_{d-h}^{J_{d-h}}\left(u1\right)}\\ \; & =\varepsilon_{m}\;O\left(1\right), \end{array}$$

where the O(1)-term does not depend on *u*. Thus, one can conclude that condition (20) of Proposition 5 is satisfied. □

An analogous result as the one stated in Proposition 5 can be stated for $\lambda_{U}$.

### 3.3. Schmid’s and Schmidt’s Tail Dependence Measure

Schmid and Schmidt (see [5] (Sec. 3.3)) considered a generalization of tail coefficients based on a multivariate conditional version of Spearman’s rho, which is defined as

$$\begin{array}{c} \rho \left(C_{d},g\right)=\frac{\int_{{\left[0,1\right]}^{d}}C_{d}\left(u\right)g\left(u\right)du-\int_{{\left[0,1\right]}^{d}}\Pi_{d}\left(u\right)g\left(u\right)du}{\int_{{\left[0,1\right]}^{d}}M_{d}\left(u\right)g\left(u\right)du-\int_{{\left[0,1\right]}^{d}}\Pi_{d}\left(u\right)g\left(u\right)du} \end{array}$$

for some non-negative measurable function *g* provided that the integrals exist. The choice $g\left(u\right)=𝟙(u\in {\left[0,p\right]}^{d})$ leads to

$$\begin{array}{c} \rho_{1}\left(C_{d},p\right)=\frac{\int_{{\left[0,p\right]}^{d}}C_{d}\left(u\right)du-\int_{{\left[0,p\right]}^{d}}\Pi_{d}\left(u\right)du}{\int_{{\left[0,p\right]}^{d}}M_{d}\left(u\right)du-\int_{{\left[0,p\right]}^{d}}\Pi_{d}\left(u\right)du} \end{array}$$

and the multivariate tail dependence measure is defined as

(24) $$\begin{array}{c} \lambda_{L,S}\left(C_{d}\right)=\lim_{p↘0}\rho_{1}\left(C_{d},p\right)=\lim_{p↘0}\frac{d+1}{p^{d+1}}\int_{{\left[0,p\right]}^{d}}C_{d}\left(u\right)du, \end{array}$$

provided the existence of the limit. Similarly, they define

(25) $$\begin{array}{c} \lambda_{U,S}\left(C_{d}\right)=\lim_{p↘0}\frac{\int_{{\left[1-p,1\right]}^{d}}C_{d}\left(u\right)du-\int_{{\left[1-p,1\right]}^{d}}\Pi_{d}\left(u\right)du}{\int_{{\left[1-p,1\right]}^{d}}M_{d}\left(u\right)du-\int_{{\left[1-p,1\right]}^{d}}\Pi_{d}\left(u\right)du}. \end{array}$$

Additionally, these coefficients are not equal to $\lambda_{L},\lambda_{U}$, respectively, in the bivariate case, so we can consider it more as a different type of tail dependence coefficient rather than a generalization.

**Proposition** **6.** *Schmid’s and Schmidt’s tail dependence measure $\lambda_{L,S}$ satisfies normalization property $\left(T_{1}\right)$, permutation invariance property $\left(T_{3}\right)$, and addition property $\left(T_{4}^{\prime }\right)$, with $k_{d}\left(\lambda_{L,S}\right)=0$ for every d≥2.*

**Proof.**  Normalization property $\left(T_{1}\right)$ and permutation invariance $\left(T_{3}\right)$ follow from the normalization property and permutation invariance of Spearman’s rho, see, for example [16]. When adding an independent component, one gets

$$\begin{array}{c} \lambda_{L,S}\left(C_{d+1}\right)=\lim_{p↘0}\frac{d+2}{p^{d+2}}\int_{{\left[0,p\right]}^{d+1}}C_{d}\left(u\right)udu=\lim_{p↘0}\frac{p(d+2)}{2(d+1)}\frac{d+1}{p^{d+1}}\int_{{\left[0,p\right]}^{d}}C_{d}\left(u\right)du=0. \end{array}$$

This finishes the proof. □

In order for duality property $\left(T_{5}\right)$ to hold, the upper version should rather be defined as

(26) $$\begin{array}{c} \lambda_{U,S}^{*}\left(C_{d}\right)=\lim_{p↘0}\frac{d+1}{p^{d+1}}\int_{{\left[0,p\right]}^{d}}C_{d}^{S}\left(u\right)du. \end{array}$$

This seems to be more logical, since $\lambda_{U,S}\left(C_{d}\right)$ can only be expressed, after substituting

(27) $$\int_{{\left[1-p,1\right]}^{d}}\Pi_{d}\left(u\right)du={\left[\frac{p(2-p)}{2}\right]}^{d}\;\mathrm{and}\;\int_{{\left[1-p,1\right]}^{d}}M_{d}\left(u\right)du=p^{d}-\frac{d}{d+1}p^{d+1}$$

into (25), as

$$\begin{array}{c} \lambda_{U,S}\left(C_{d}\right)=\lim_{p↘0}\frac{\int_{{\left[1-p,1\right]}^{d}}C_{d}\left(u\right)du-{\left[\frac{p(2-p)}{2}\right]}^{d}}{p^{d}-\frac{d}{d+1}p^{d+1}-{\left[\frac{p(2-p)}{2}\right]}^{d}} \end{array}$$

which cannot be further simplified. It is easy to show that in the bivariate case (i.e., d=2) the coefficients $\lambda_{U,S}\left(C_{d}\right)$ and $\lambda_{U,S}^{*}\left(C_{d}\right)$ coincide. For a general dimension d>2 however they can differ.

The continuity property $\left(T_{2}\right)$ cannot be shown in full generality, but a continuity property is fulfilled in the special case of a sequence of contaminated copulas, as in (14).

**Proposition** **7.** *Consider a sequence of contaminated copulas, $C_{d,m}=\left(1-\epsilon_{m}\right)C_{d}+\epsilon_{m}B_{d,m}$, such that $\epsilon_{m}\to 0$, as m→∞, and $\lambda_{L,S}\left(C_{d}\right)$ exists. Afterwards, as m→∞,*

$$\begin{array}{c} \lambda_{L,S}\left(C_{d,m}\right)\to \lambda_{L,S}\left(C_{d}\right). \end{array}$$

**Proof.**  Direct calculation gives

$$\begin{array}{c} \lim_{m\to \infty }\lambda_{L,S}\left(C_{d,m}\right)=\lim_{m\to \infty }\left[\left(1-\epsilon_{m}\right)\lambda_{L,S}\left(C_{d}\right)+\epsilon_{m}\lambda_{L,S}\left(B_{d,m}\right)\right]=\lambda_{L,S}\left(C_{d}\right) \end{array}$$

since $\lambda_{L,S}\left(B_{d,m}\right)$ is bounded. □

### 3.4. Tail Dependence of Extreme-Value Copulas

As stated in (6), bivariate tail coefficients for extreme-value copulas can be simply expressed using the corresponding Pickands dependence function. Thus tail dependence is fully determined by the Pickands dependence function $A_{2}$ in the point (1/2,1/2). The range of values for $A_{2}$ is limited by $\max\left(w_{1},w_{2}\right)\leq A_{2}\left(w_{1},w_{2}\right)\leq 1$, which also gives us $1/2\leq A_{2}\left(1/2,1/2\right)\leq 1$ where the bivariate tail coefficient $\lambda_{U}$ gets larger when $A_{2}\left(1/2,1/2\right)$ is closer to 1/2. On the other hand, $A_{2}\left(1/2,1/2\right)=1$ means tail independence. Following this idea and given that also for the *d*-dimensional Pickands dependence function $A_{d}$ associated to a copula $C_{d}$ we have $1/d\leq A_{d}\left(1/d,\cdots ,1/d\right)\leq 1$, a measure of tail dependence for *d*-dimensional extreme-value copulas could be based on the difference $1-A_{d}\left(1/d,\cdots ,1/d\right)$. After proper standardization, this leads to

(28) $$\begin{array}{c} \lambda_{U,E}\left(C_{d}\right)=\frac{d}{d-1}\left(1-A_{d}\left(1/d,\cdots ,1/d\right)\right). \end{array}$$

Note that such a coefficient is equal to a translation of the extremal coefficient given in [17] or [7] and defined as $\theta_{E}=d\cdot A_{d}\left(1/d,\cdots ,1/d\right)$. This coefficient $\theta_{E}$ was termed extremal coefficient in [17]. Schlather and Town (see [18]) give a simple interpretation of $\theta_{E}$, related to the amount of independent variables that are involved in the corresponding *d*-variate random vector.

**Proposition** **8.** *The multivariate tail dependence coefficient $\lambda_{U,E}$ in *(28)* satisfies normalization property $\left(T_{1}\right)$, continuity property $\left(T_{2}\right)$, permutation invariance property $\left(T_{3}\right)$, and addition property $\left(T_{4}^{\prime }\right)$, with $k_{d}\left(\lambda_{U,E}\right)=\frac{d-1}{d}$ for every d≥2.*

**Proof.**  Normalization $\left(T_{1}\right)$ and permutation invariance $\left(T_{3}\right)$ follow immediately from the definition of $\lambda_{U,E}$. If $\lim_{m\to \infty }C_{d,m}\left(u\right)=C_{d}\left(u\right),\forall u\in {\left[0,1\right]}^{d}$, and then also $\lim_{m\to \infty }A_{d,m}\left(w\right)=A_{d}\left(w\right),\forall w\in \Delta_{d-1}$, which proves the validity of $\left(T_{2}\right)$. For $X_{d+1}$ independent of $\left(X_{1},\cdots ,X_{d}\right)$, we can use Example 1 and obtain

$$\begin{array}{cc} \lambda_{U,E}\left(C_{d+1}\right)=\frac{d+1}{d}\left(1-A_{d+1}\left(\frac{1}{d+1},\cdots ,\frac{1}{d+1}\right)\right)\; & =\frac{d+1}{d}\left(1-\frac{1}{d+1}\left(dA_{d}\left(\frac{1}{d},\cdots ,\frac{1}{d}\right)+1\right)\right)\\ \; & =1-A_{d}\left(\frac{1}{d},\cdots ,\frac{1}{d}\right)\\ \; & =\frac{d-1}{d}\lambda_{U,E}\left(C_{d}\right). \end{array}$$

 □

**Remark** **1.** *The duality property $\left(T_{5}\right)$ is not applicable, since the survival copula of an extreme-value copula does not have to be an extreme-value copula.*

### 3.5. Tail Dependence Using Subvectors

A common element of the multivariate tail dependence measures discussed in Section 3.1, Section 3.2 and Section 3.3 is that they focus on extremal behavior of all *d* components of a random vector X. However, one could also be interested in knowing whether there is any kind of tail dependence present in the vector, which is even for subvectors of X. An interesting observation to be made is for tail dependence measures that satisfy property $\left(T_{4}^{\prime }\right)$ with $k_{d}=0$ for every d≥2. Assume that *X* and *Y* are independent random variables. Then any tail measure ${𝓉}_{2}\left(C_{2}\right)$ would be zero for the random couple (X,Y) and no matter which random component we add the tail measure for the extended random vector would stay 0. In other words, for any such tail dependence measure, this leads to tail independence of the *d*-dimensional random vector ${\left(X,\cdots ,X,Y\right)}^{⊤}$, no matter what *d* is. Considering tail dependence of subvectors would be of particular interest in this case.

Suppose that we have a multivariate tail coefficient $\mu_{L,d}$ that can be calculated for general dimension d≥2. Suppose further that this coefficient only depends on the strength of tail dependence when all of the components of a random vector are simultaneously large or small. This is the case for all multivariate tail coefficients mentioned in Section 3.1, Section 3.2 and Section 3.3. Subsequently, we can introduce a tail coefficient given by

(29) μL(Cd)=∑j=2dwd,j∑1≤ℓ1<⋯<ℓj≤dμL,j(C(ℓ1,⋯,ℓj))=∑j=2dw˜d,j1dj∑1≤ℓ1<⋯<ℓj≤dμL,j(C(ℓ1,⋯,ℓj))

where 1dj∑1≤ℓ1<⋯<ℓj≤dμL,j(C(ℓ1,⋯,ℓj)) can be interpreted as an average tail dependence measure per dimension, and where w˜d,j=wd,jdj. This measure deals with a disadvantage of current multivariate tail coefficients that assign a value of 0 to the copulas, where d−1 components are highly dependent in their tail, and the *d*-th component is independent. When dealing with possible stock losses, for example, this situation should be also captured by a tail coefficient.

Recall that the weight ${\tilde{w}}_{d,j}$ corresponds to an importance given to the average tail dependence within all the *j*-dimensional subvectors of X. Because tail dependence in a higher dimension is more severe, as more extremes occur simultaneously, it is natural to assume ${\tilde{w}}_{d,2}\leq {\tilde{w}}_{d,3}\leq \cdots \leq {\tilde{w}}_{d,d}$. However, such an assumption excludes other approaches to measure tail dependence. For example, setting ${\tilde{w}}_{d,2}=1$ and ${\tilde{w}}_{d,j}=0$ for j=3,⋯,d would lead to the construction of a tail dependence measure as the average of all pairwise measures. If the underlying bivariate measure satisfies $\left(T_{1}\right)$, $\left(T_{2}\right)$, $\left(T_{3}\right)$, and $\left(T_{5}\right)$ with d=2 only, these properties are carried over to the pairwise measure. Additionally, $\left(T_{4}^{\prime }\right)$ can be shown similarly as in Proposition 1 in [16]. Despite possibly fulfilling the desirable properties, all of the higher dimensional dependencies are ignored, being a clear drawback of such a pairwise approach. In the sequel, we focus on the setting that ${\tilde{w}}_{d,2}\leq {\tilde{w}}_{d,3}\leq \cdots \leq {\tilde{w}}_{d,d}$.

**Proposition** **9.** *Suppose that the tail dependence measures $\mu_{L,j}$ satisfy normalization property $\left(T_{1}\right)$, continuity property $\left(T_{2}\right)$, permutation invariance property $\left(T_{3}\right)$, and duality property $\left(T_{5}\right)$, for j=2,⋯,d. Further assume that $\sum_{j=2}^{d}{\tilde{w}}_{d,j}=1$. Subsequently, the coefficient $\mu_{L}$ in *(29)* also satisfies properties $\left(T_{1}\right)$, $\left(T_{2}\right)$, $\left(T_{3}\right)$, and $\left(T_{5}\right)$.*

**Proof.**  Clearly $\mu_{L}\left(\Pi_{d}\right)=0$ and $\mu_{L}\left(M_{d}\right)=\sum_{j=2}^{d}{\tilde{w}}_{d,j}=1$. The continuity, permutation invariance, and duality properties follow from the continuity, permutation invariance, and duality properties of $\mu_{L,j}$. □

What happens in case of the addition of an independent component (property $\left(T_{4}\right)$) is not so straightforward, since the weights differ depending on the overall dimension *d*. The addition of an independent component increases dimension and, thus, possibly changes all of the weights. However, one could try to come up with a weighting scheme that guarantees fulfilment of property $\left(T_{4}\right)$. Consider $X_{d+1}$ independent of ${\left(X_{1},\cdots ,X_{d}\right)}^{⊤}$. Suppose that the input tail dependence measures $\mu_{L,j}$ satisfy property $\left(T_{4}^{\prime }\right)$, with $k_{j}=k_{j}\left(\mu_{L,j}\right)$ for j=2,⋯,d. First, we express $\mu_{L}$ for the random vector ${\left(X_{1},\cdots ,X_{d+1}\right)}^{⊤}$, as

(30) μL(Cd+1)=∑j=2d+1w˜d+1,j1d+1j∑1≤ℓ1<⋯<ℓj≤d+1μL,j(Cj(ℓ1,⋯,ℓj))=∑j=2dw˜d+1,j1d+1j∑1≤ℓ1<⋯<ℓj≤dμL,j(Cj(ℓ1,⋯,ℓj))=+∑j=2d+1w˜d+1,j1d+1j∑1≤ℓ1<⋯<ℓj−1≤dμL,j(Cj(ℓ1,⋯,ℓj−1,d+1)).

Now using property $\left(T_{4}^{\prime }\right)$ in (30) together with the fact that for index j=2, the corresponding summand is $\mu_{L,2}\left(\Pi_{2}\right)=0$ and, thus, this index can be omitted, one obtains

μL(Cd+1)=∑j=2dw˜d+1,jd+1−jd+11dj∑1≤ℓ1<⋯<ℓj≤dμL,j(Cj(ℓ1,⋯,ℓj))=+∑j=3d+1w˜d+1,jkj−1d+1j∑1≤ℓ1<⋯<ℓj−1≤dμL,j−1(Cj−1(ℓ1,⋯,ℓj−1))=∑j=2dw˜d+1,jd+1−jd+1+w˜d+1,j+1kjj+1d+11dj∑1≤ℓ1<⋯<ℓj≤dμL,j(Cj(ℓ1,⋯,ℓj))

which is equal to $\mu_{L}\left(C_{d}\right)$ with weights given as

$${\tilde{w}}_{d,j}={\tilde{w}}_{d+1,j}\frac{d+1-j}{d+1}+{\tilde{w}}_{d+1,j+1}k_{j}\frac{j+1}{d+1}$$

for every j=2,⋯,d. A sufficient criterion for fulfillment of property $\left(T_{4}\right)$ would thus be to have

(31) $${\tilde{w}}_{d,j}\geq {\tilde{w}}_{d+1,j}\frac{d+1-j}{d+1}+{\tilde{w}}_{d+1,j+1}k_{j}\frac{j+1}{d+1}$$

for every j=2,⋯,d. Knowing the values $k_{j}$, ${\tilde{w}}_{d,j}$, ${\tilde{w}}_{d+1,j}$, for j=2,⋯,d, and ${\tilde{w}}_{d+1,d+1}$, one can check (31).

One rather general method of weight selection can then be as follows. Suppose that one wants to achieve that proportions of weights $w_{d,d_{1}}$ and $w_{d,d_{2}}$ corresponding to two subdimensions $d_{1}$ and $d_{2}$ do not depend on the overall dimension *d*. This can be achieved by setting recursively $w_{d+1,j}=r_{d+1}w_{d,j}$ for j=2,⋯,d and $w_{d+1,d+1}=1-\sum_{j=2}^{d}w_{d+1,j}=1-r_{d+1}$. The initial condition is obviously given as $w_{2,2}=1$. To obtain ${\tilde{w}}_{d,2}\leq {\tilde{w}}_{d,3}\leq \cdots \leq {\tilde{w}}_{d,d}$, one needs that $r_{d}\in \left[0,1/2\right]$ for every d=3,⋯. Values of $r_{d}$ closer to 0 give more weight to the *d*-th dimension, values close to 1/2 limit its influence. If we further assume that $r_{d}=r$, which is $r_{d}$ does not depend on *d*, this further simplifies to

$$w_{d,j}=r^{d-j}{\left(1-r\right)}^{𝟙\{j>2\}}$$

for d=2,⋯ and j=2,⋯,d. We next check the condition in (31) for this particular weight selection. Condition (31) can be rewritten as

(32) $$\begin{array}{c} 1\geq r\frac{d+1-j}{d+1}+k_{j}\frac{j+1}{d+1},\;\mathrm{for}\;\mathrm{every}\;j=2,\ldots ,d. \end{array}$$

If $k_{j}=1$ for every *j* as in one case of Li’s tail dependence parameter, condition (32) allows for only one selection of *r*, which is r=0. On the other hand, if $k_{j}=0$ for every *j*, *r* can take any values in [0,1/2]. Looking from the other perspective, if r=1/2, then condition (32) is satisfied if

$$\begin{array}{c} k_{j}\leq \frac{d+1/2}{d+1},\;\mathrm{for}\;\mathrm{every}\;j=2,\ldots ,d. \end{array}$$

Let us recall that these conditions can only be seen as sufficient, not necessary. A precise study of what happens when an independent component is added requires knowledge of the weighting scheme and knowledge of the underlying input tail dependence measure.

In summary, the above discussion reveals that a measure that is able to detect tail dependence not only in a random vector as a whole, but also in lower-dimensional subvectors, can be constructed. A simple and interpretable weighting scheme proposed above can be used, such that several desirable properties of the tail dependence measure are guaranteed.

### 3.6. Overview of Multivariate Tail Coefficients and Properties

For convenience of the reader, we list in Table 1 all of the discussed tail dependence measures, with reference to their section number, and indicate which properties they satisfy.

## 4. Multivariate Tail Coefficients: Further Properties

In Section 3, the focus was on properties $\left(T_{1}\right)$–$\left(T_{5}\right)$. In this section, we aim at exploring some further properties that might be of special interest. We, in particular, investigate the following type of properties. Here, ${𝓉}_{d}\left(C_{d}\right)$ denotes a multivariate tail coefficient for $C_{d}\in \mathrm{Cop}\left(d\right)$. When needed, we specify whether it concerns a lower or upper tail coefficient, referring to them as ${𝓉}_{L,d}\left(C_{d}\right)$ and ${𝓉}_{U,d}\left(C_{d}\right)$, respectively.

- *Expansion property ($P_{1}$)*.
  Given is a random vector $X={\left(X_{1},\cdots ,X_{d}\right)}^{⊤}$ with copula $C_{d}$. One adds one random component $X_{d+1}$ to X. Denote the copula of the expanded random vector ${\left(X^{⊤},X_{d+1}\right)}^{⊤}$ by $C_{d+1}$. How does ${𝓉}_{d+1}\left(C_{d+1}\right)$ compare to ${𝓉}_{d}\left(C_{d}\right)$? Does it hold that ${𝓉}_{d+1}\left(C_{d+1}\right)\leq {𝓉}_{d}\left(C_{d}\right)$?
- *Monotonicity property ($P_{2}$)*.
  Consider two copulas $C_{d,1},C_{d,2}\in \mathrm{Cop}\left(d\right)$. Does the following hold?
  (i) If $C_{d,1}\left(u\right)\leq C_{d,2}\left(u\right)$ for u in some neighborhood of 0, then ${𝓉}_{L,d}\left(C_{d,1}\right)\leq {𝓉}_{L,d}\left(C_{d,2}\right)$.
  (ii) If ${\bar{C}}_{d,1}\left(u\right)\leq {\bar{C}}_{d,2}\left(u\right)$ for u in some neighborhood of 1, then ${𝓉}_{U,d}\left(C_{d,1}\right)\leq {𝓉}_{U,d}\left(C_{d,2}\right)$.
- *Convex combination property ($P_{3}$)*.
  Suppose that the copula $C_{d}$ can be written as $C_{d}=\alpha C_{d,1}+\left(1-\alpha \right)C_{d,2}$ for α∈[0,1], and $C_{d,1},C_{d,2}\in \mathrm{Cop}\left(d\right)$. What can we say about the comparison between ${𝓉}_{d}\left(C_{d}\right)$ and $\alpha {𝓉}_{d}\left(C_{d,1}\right)+\left(1-\alpha \right){𝓉}_{d}\left(C_{d,2}\right)$?
  For extreme-value copulas, we look into geometric combinations instead.

The logic behind property $\left(P_{1}\right)$ comes from the perception of a tail coefficient as a probability of extreme events of components of the random vector to happen simultaneously. Thus, when another component is added, the probability of having extreme events cannot increase. However, there is no such a limitation from below and adding a component can immediately lead to a decrease of the coefficient to zero.

In the next subsections, we briefly discuss these properties for the multivariate tail coefficients discussed in Section 3.

### 4.1. Expansion Property (P_1_)

For Frahm’s coefficient, it holds that $\epsilon_{L}\left(C_{d+1}\right)\leq \epsilon_{L}\left(C_{d}\right)$ and analogously for the upper coefficient. This result can be found in Proposition 2 of [3].

For Li’s tail dependence parameters, we need to distinguish two cases. If we add the new component to the set $I_{h}$, then we have

$$\begin{array}{c} \lambda_{L}^{I_{h+1}|J_{d-h}}\left(C_{d+1}\right)=\lim_{u↘0}\frac{C_{d+1}\left(u1\right)}{C_{d-h}^{J_{d-h}}\left(u1\right)}\leq \lim_{u↘0}\frac{C_{d}\left(u1\right)}{C_{d-h}^{J_{d-h}}\left(u1\right)}=\lambda_{L}^{I_{h}|J_{d-h}}\left(C_{d}\right). \end{array}$$

However, if the component is added to the set $J_{d-h}$, no relationship can be shown, in general. A special situation occurs when the component $X_{d+1}$ added to the set $J_{d-h}$ is just a duplicate of a component, which is already included in $J_{d-h}$. Subsequently, obviously $\lambda_{L}^{I_{h}|J_{d-h+1}}\left(C_{d+1}\right)=\lambda_{L}^{I_{h}|J_{d-h}}\left(C_{d}\right)$.

For Schmid’s and Schmidt’s tail dependence measures, one cannot say, in general, how the coefficient $\lambda_{L,S}\left(C_{d+1}\right)$ behaves when compared to $\lambda_{L,S}\left(C_{d}\right)$. As can be seen from (24), the integral expression decreases with increasing dimension *d*, but, at the same time, the normalizing constant increases with *d*.

For the tail coefficient for extreme-value copulas, $\lambda_{U,E}\left(C_{d}\right)$ it follows from Example 7 in Section 6 that the addition of another component can lead to an increase in this coefficient. See, in particular, also Figure 5.

### 4.2. Monotonicity Property (P_2_)

Concerning the monotonicity property $\left(P_{2}\right)$ it is easily seen that $\left(P_{2}\right)$(i) holds for Frahm’s lower dependence coefficient $\epsilon_{L}\left(C_{d}\right)$ if we additionally assume that ${\bar{C}}_{d,1}\left(u\right)\leq {\bar{C}}_{d,2}\left(u\right)$ for u in some neighborhood of 0. Similarly, we need to assume that $C_{d,1}\left(u\right)\leq C_{d,2}\left(u\right)$ for u in some neighborhood of 1 in order to show that $\left(P_{2}\right)$ (ii) holds.

For Li’s tail dependence parameters, property $\left(P_{2}\right)$ does not hold in general. This is illustrated via the following example in case d=4. Consider a random vector ${\left(U_{1},U_{2},U_{3},U_{4}\right)}^{⊤}$ with uniform marginals and with distribution function a Clayton copula with parameter θ>0 (see Example 6), given by $C_{4,1}\left(u\right)={\left(u_{1}+u_{2}+u_{3}+u_{4}-3\right)}^{1/\theta }$ (see (39)). We denote this first copula by $C_{4,1}$. Note that the random vector ${\left(U_{1},U_{2},U_{3}\right)}^{⊤}$ has as joint distribution a three-dimensional Clayton copula with parameter θ, which we denote by $C_{3}$. The vector ${\left(U_{1},U_{2},U_{4}\right)}^{⊤}$ has the same joint distribution $C_{3}$. Next, we consider the copula of the random vector ${\left(U_{1},U_{2},U_{3},U_{3}\right)}^{⊤}$ that we denote by $C_{4,2}$. One has that, for all $u\in {\left[0,1\right]}^{4}$,

$$\begin{array}{ccc} C_{4,1}\left(u\right) & = & P\left(U_{1}\leq u_{1},U_{2}\leq u_{2},U_{3}\leq u_{3},U_{4}\leq u_{4}\right)\\ & \leq & \min\left(P\left(U_{1}\leq u_{1},U_{2}\leq u_{2},U_{3}\leq u_{3}\right),P\left(U_{1}\leq u_{1},U_{2}\leq u_{2},U_{4}\leq u_{4}\right)\right)\\ & = & \min\left(C_{3}\left(u_{1},u_{2},u_{3}\right),C_{3}\left(u_{1},u_{2},u_{4}\right)\right)\\ & = & C_{3}\left(u_{1},u_{2},\min\left(u_{3},u_{4}\right)\right)\\ & = & C_{4,2}\left(u\right). \end{array}$$

In Example 6 we calculate Li’s lower tail dependence parameter for a *d*-variate Clayton copula, which equals $\lambda_{L}^{I_{h}|J_{d-h}}\left(C_{d}\right)={\left((d-h)/d\right)}^{1/\theta }$ (see (41)). Applying this in the setting of the current example leads to

$$\lambda_{L}^{1,2|3,4}\left(C_{4,2}\right)=\lambda_{L}^{1,2|3}\left(C_{3}\right)={\left(\frac{1}{3}\right)}^{1/\theta }<{\left(\frac{2}{4}\right)}^{1/\theta }=\lambda_{L}^{1,2|3,4}\left(C_{4,1}\right),$$

which thus contradicts monotonicity property $\left(P_{2}\right)$(i).

From the definition of Schmid’s and Schmidt’s tail dependence measure, it is immediate that the monotonicity property $\left(P_{2}\right)$ holds.

For the tail coefficient for extreme-value copulas, $\lambda_{U,E}$ defined in (28) the monotonicity property $\left(P_{2}\right)$ holds. To see this, recall from (3), that, for an extreme-value copula $C_{d,1}$, we can express its stable tail dependence function as

(33) $$\ell_{C_{d,1}}\left(x_{1},\cdots ,x_{d}\right)=-\log\left(C_{d,1}\left(e^{-x_{1}},\cdots ,e^{-x_{d}}\right)\right),$$

and, hence, using that $C_{d,1}\leq C_{d,2}$, it follows that $\ell_{C_{d,1}}\geq \ell_{C_{d,2}}$. The same inequality holds for Pickands dependence function $A_{d,1}$, which is a restriction of the stable tail dependence function $\ell_{C_{d,1}}$ on the unit simplex. Hence, $C_{d,1}\leq C_{d,2}$ also implies that $A_{C_{d,1}}\geq A_{C_{d,2}}$. From the definition of the tail coefficient in (28) it thus follows $\lambda_{U,E}\left(C_{d,1}\right)\leq \lambda_{U,E}\left(C_{d,2}\right)$.

### 4.3. Investigation of a Tail Coefficient for a Convex/Geometric Combination (Property (P_3_))

Consider a copula $C_{d}$ that is a convex combination of two copulas $C_{d,1}$ and $C_{d,2}$, i.e., $C_{d}=\alpha C_{d,1}+\left(1-\alpha \right)C_{d,2}$ for α∈[0,1]. For the survival function, we then also have ${\bar{C}}_{d}=\alpha {\bar{C}}_{d,1}+\left(1-\alpha \right){\bar{C}}_{d,1}$.

Before stating the results for the various multivariate tail coefficients, we first make the following observation. For α,a,b,c,d∈[0,1], it is straightforward to show that

(34) $$\begin{array}{c} \frac{a}{c}\leq \frac{\alpha a+(1-\alpha )b}{\alpha c+(1-\alpha )d}\leq \frac{b}{d}⟺\frac{a}{c}\leq \frac{b}{d}. \end{array}$$

Frahm’s lower extremal dependence coefficient for the copula $C_{d}$ is given by

$$\begin{array}{c} \epsilon_{L}\left(C_{d}\right)=\lim_{u↘0}\frac{\alpha C_{d,1}\left(u1\right)+\left(1-\alpha \right)C_{d,2}\left(u1\right)}{\alpha \left(1-{\bar{C}}_{d,1}\left(u1\right)\right)+\left(1-\alpha \right)\left(1-{\bar{C}}_{d,2}\left(u1\right)\right)}. \end{array}$$

Using (34), it then follows that, if $\epsilon_{L}\left(C_{d,1}\right)\leq \epsilon_{L}\left(C_{d,2}\right)$, then

$$\epsilon_{L}\left(C_{d,1}\right)\leq \epsilon_{L}\left(C_{d}\right)\leq \epsilon_{L}\left(C_{d,2}\right).$$

The same conclusion can be found for Frahm’s upper extremal dependence coefficient $\epsilon_{U}$.

Li’s lower tail dependence parameter for $C_{d}$, a convex mixture of copulas, equals

$$\begin{array}{c} \lambda_{L}^{I_{h}|J_{d-h}}\left(C_{d}\right)=\lim_{u↘0}\frac{\alpha C_{d,1}\left(u1\right)+\left(1-\alpha \right)C_{d,2}\left(u1\right)}{\alpha C_{d-h,1}^{J_{d-h}}\left(u1\right)+\left(1-\alpha \right)C_{d-h,2}^{J_{d-h}}}, \end{array}$$

and an application of (34) gives that, if $\lambda_{L}^{I_{h}|J_{d-h}}\left(C_{d,1}\right)\leq \lambda_{L}^{I_{h}|J_{d-h}}\left(C_{d,2}\right)$, then $\lambda_{L}^{I_{h}|J_{d-h}}\left(C_{d,1}\right)\leq \lambda_{L}^{I_{h}|J_{d-h}}\left(C_{d}\right)\leq \lambda_{L}^{I_{h}|J_{d-h}}\left(C_{d,2}\right)$. The same conclusion can be found for Li’s upper tail dependence parameter $\lambda_{U}^{I_{h}|J_{d-h}}$.

Schmid’s and Schmidt’s lower tail dependence measure for a convex mixture of copulas is

$$\begin{array}{c} \lambda_{L,S}\left(C_{d}\right)=\lim_{p↘0}\frac{d+1}{p^{d+1}}\int_{{\left[0,p\right]}^{d}}\left[\alpha C_{d,1}\left(u\right)+\left(1-\alpha \right)C_{d,2}\left(u\right)\right]du=\alpha \lambda_{L,S}\left(C_{d,1}\right)+\left(1-\alpha \right)\lambda_{L,S}\left(C_{d,2}\right). \end{array}$$

For an extreme-value copula, it does not make sense to look at convex combinations of two extreme-value copulas, since it cannot be shown, in general, that such a convex combination would again be an extreme-value copula. A more natural way to combine two extreme-value copulas $C_{d,1}$ and $C_{d,2}$ is by means of a geometric combination, i.e., by considering $C_{d}=C_{d,1}^{\alpha }C_{d,2}^{1-\alpha }$, with α∈[0,1]. In, for example, Falk et al. [19] (p. 123) it was shown that a convex combination of two Pickands dependence functions is also a Pickands dependence function. Denoting by $A_{d,1}$ and $A_{d,2}$, the Pickands dependence functions of $C_{d,1}$ and $C_{d,2}$, respectively, it then follows from (33) that the Pickands dependence function $A_{d}$ for $C_{d}=C_{d,1}^{\alpha }C_{d,2}^{1-\alpha }$, is given by $A_{d}=\alpha A_{d,1}+\left(1-\alpha \right)A_{d,2}$. From this it is seen that $C_{d}$ is again an extreme-value copula. For the tail dependence coefficient for extreme-value copulas, it thus holds that

$$\begin{array}{cc} \lambda_{U,E}\left(C_{d}\right) & =\frac{d}{d-1}\left(1-\alpha A_{d,1}\left(1/d,\cdots ,1/d\right)-\left(1-\alpha \right)A_{d,2}\left(1/d,\cdots ,1/d\right)\right)\\ \; & =\alpha \lambda_{U,E}\left(C_{d,1}\right)+\left(1-\alpha \right)\lambda_{U,E}\left(C_{d,2}\right), \end{array}$$

i.e., the coefficient $\lambda_{U,E}$ of a geometric mean of two extreme-value copulas is equal to the corresponding convex combination of the coefficients of the concerned two copulas.

## 5. Tail Coefficients for Archimedean Copulas in Increasing Dimension

A natural question to examine is an influence of increasing dimension on possible multivariate tail dependence. If one restricts to the class of Archimedean copulas, several results can be achieved, despite that similar problems with interchanging limits occur while studying the continuity property $\left(T_{2}\right)$. First, let us formulate a useful lemma that describes the behavior of the main diagonal of Archimedean copulas when the dimension increases.

**Lemma** **2.** *Let $\left\{C_{d}\right\}$ be a sequence of d-dimensional Archimedean copulas with (the same) generator ψ. Then for u∈[0,1) and v∈(0,1]*

$$\begin{array}{c} \lim_{d\to \infty }C_{d}\left(u,\cdots ,u\right)=0,\\ \lim_{d\to \infty }{\bar{C}}_{d}\left(v,\cdots ,v\right)=0. \end{array}$$

**Proof.**  The proof is along the same lines as the proof of Proposition 9 in [16]. □

This lemma can be used in the following statements that focus on individual multivariate tail coefficients. The first one to be examined is the Frahm’s extremal dependence coefficient $\epsilon_{L}$.

**Proposition** **10.** *Let $\left\{C_{d}\right\}$ be a sequence of d-dimensional Archimedean copulas with (the same) generator ψ. Further assume that*

$$\begin{array}{c} \lim_{d\to \infty }\lim_{u↘0}\frac{C_{d}\left(u1\right)}{1-{\bar{C}}_{d}\left(u1\right)}=\lim_{u↘0}\lim_{d\to \infty }\frac{C_{d}\left(u1\right)}{1-{\bar{C}}_{d}\left(u1\right)}. \end{array}$$

*Then*

$$\begin{array}{c} \lim_{d\to \infty }\epsilon_{L}\left(C_{d}\right)=0. \end{array}$$

**Proof.**  The statement follows by the direct application of Lemma 2, since then

$$\begin{array}{c} \lim_{d\to \infty }\epsilon_{L}\left(C_{d}\right)=\lim_{u↘0}\lim_{d\to \infty }\frac{C_{d}\left(u1\right)}{1-{\bar{C}}_{d}\left(u1\right)}=0. \end{array}$$

 □

An analogous result could be stated for $\epsilon_{U}$.

**Remark** **2.** *The condition on interchanging limits is, in general, difficult to check. However, we discuss some examples in which the condition can be checked. A first example is that of the independence copula $C_{d}\left(u\right)=\Pi \left(u\right)$ for which $C_{d}\left(u1\right)=u^{d}$ and ${\bar{C}}_{d}\left(u1\right)={\left(1-u\right)}^{d}$. Henceforth, $\lim_{u↘0}\frac{C_{d}\left(u1\right)}{1-{\bar{C}}_{d}\left(u1\right)}=0$ for all u∈[0,1]. Furthermore, $\lim_{d\to \infty }\frac{C_{d}\left(u1\right)}{1-{\bar{C}}_{d}\left(u1\right)}=0$, for all u∈[0,1). Consequently, in this example, the condition of interchanging limits holds. A second example is the Gumbel–Hougaard copula also considered in Example 7 in Section 6. For this copula it can be seen that, as in the previous example, the two concerned limits (when u→0 and when d→∞) are zero and, hence, interchanging the limits is also valid in this example.*

*Proposition 10 further shows that if we construct estimators (based on values of u close to 0 or close to 1) of the limits above for Archimedean copulas in high dimensions, these will be very close to 0.*

For Li’s tail dependence parameters $\lambda_{L}^{I_{h}|J_{d-h}}$ and $\lambda_{U}^{I_{h}|J_{d-h}}$, the situation is further complicated by the necessary selection of $I_{h}$ and $J_{d-h}$ and, in particular, of the cardinality *h*. However, if the cardinality of the set $J_{d-h}$ is kept constant when the dimension *d* increases, the following result can be achieved.

**Proposition** **11.** *Let $\left\{C_{d}\right\}$ be a sequence of d-dimensional Archimedean copulas with (the same) generator ψ and let h in definition of $\lambda_{L}^{I_{h}|J_{d-h}}$ be given as $h\left(d\right)=d-h^{*}$ for a constant $h^{*}$. Further assume that*

$$\begin{array}{c} \lim_{d\to \infty }\lim_{u↘0}\frac{C_{d}\left(u1\right)}{C_{h^{*}}\left(u1\right)}=\lim_{u↘0}\lim_{d\to \infty }\frac{C_{d}\left(u1\right)}{C_{h^{*}}\left(u1\right)}. \end{array}$$

*Subsequently*

$$\begin{array}{c} \lim_{d\to \infty }\lambda_{L}^{I_{d-h^{*}}|J_{h^{*}}}\left(C_{d}\right)=0. \end{array}$$

**Proof.**  Using Lemma 2, we obtain

$$\begin{array}{c} \lim_{d\to \infty }\lambda_{L}^{I_{d-h^{*}}|J_{h^{*}}}\left(C_{d}\right)=\lim_{u↘0}\lim_{d\to \infty }\frac{C_{d}\left(u1\right)}{C_{h^{*}}\left(u1\right)}=0, \end{array}$$

from which the statement of this proposition follows. □

An analogous statement could be formulated for $\lambda_{U}$.

What can one learn from the results in this section? Archimedean copulas may be not very appropriate in high dimensions, because of their symmetry, but they are a convenient class of copulas to use. It is good to be aware though that, when the dimension increases, the tail dependence of Archimedean copulas vanishes, at least from the perspective of $\epsilon_{L}$, $\lambda_{L}^{I_{h}|J_{d-h}}$ and their upper tail counterparts.

Obtaining similar results for different classes of copulas would also be of interest, for example, for extreme-value copulas with restrictions on Pickands dependence function. However, this is complicated by the fact that, unlike Archimedean copulas, extreme-value copulas do not share a structure that could be carried through different dimensions. Some insights into this behavior are studied using the examples given in Section 6. This section includes examples on both Archimedean and extreme-value copulas, as well as examples outside these classes.

## 6. Illustrative Examples

**Example** **4.** *Farlie–Gumbel–Morgenstern copula.*

Let $C_{d}$ be a *d*-dimensional Farlie–Gumbel–Morgenstern copula defined as

(35) $$\begin{array}{c} C_{d}\left(u\right)=u_{1}u_{2}\cdots u_{d}\left[1+\sum_{j=2}^{d}\;\sum_{1\leq k_{1}<\cdots <k_{j}\leq d}\alpha_{k_{1},\cdots ,k_{j}}\left(1-u_{k_{1}}\right)\cdots \left(1-u_{k_{j}}\right),\right] \end{array}$$

where the parameters have to satisfy the following $2^{d}$ conditions

$$\begin{array}{c} 1+\sum_{j=2}^{d}\;\sum_{1\leq k_{1}<\cdots <k_{j}\leq d}\alpha_{k_{1},\cdots ,k_{j}}\epsilon_{k_{1}}\cdots \epsilon_{k_{j}}\geq 0,\;\forall \epsilon_{1},\cdots ,\epsilon_{d}\in \left\{-1,1\right\}. \end{array}$$

This copula is neither an Archimedean nor extreme-value copula.

We first consider Frahm’s extremal dependence coefficients $\epsilon_{L}$ and $\epsilon_{U}$. From (35), up to a constant $C_{d}\left(u1\right)\approx u^{d}$ when u≈0. Further, plugging (35) into (2) gives that $1-{\bar{C}}_{d}\left(u1\right)$ behaves like a polynomial $u-u^{2}+\cdots$ when u≈0. Thus,

$$\begin{array}{cc} \epsilon_{L}\left(C_{d}\right) & =\lim_{u↘0}\frac{C_{d}\left(u1\right)}{1-{\bar{C}}_{d}\left(u1\right)}=0, \end{array}$$

because the polynomial in the numerator converges to zero faster than the polynomial in the denominator. Similarly, one obtains

$$\begin{array}{cc} \epsilon_{U}\left(C_{d}\right) & =\lim_{u↗1}\frac{{\bar{C}}_{d}\left(u1\right)}{1-C_{d}\left(u1\right)}=0. \end{array}$$

While examining $\lambda_{L}^{I_{h}|J_{d-h}}$ and $\lambda_{U}^{I_{h}|J_{d-h}}$, the very same arguments are of use. No matter how one chooses index sets $I_{h}$ and $J_{d-h}$,

$$\begin{array}{c} \lambda_{L}^{I_{h}|J_{d-h}}\left(C_{d}\right)=\lambda_{U}^{I_{h}|J_{d-h}}\left(C_{d}\right)=0 \end{array}$$

since, again, the corresponding limits contain ratios of polynomials, such that the polynomials in the numerators converge to zero faster than the polynomials in the denominators.

To obtain $\lambda_{L,S}$, the integral $\int_{{\left[0,p\right]}^{d}}C_{d}\left(u\right)du$ needs to be calculated. Consider now a special case when the only non-zero parameter is $\alpha =\alpha_{1,\cdots ,d}$. Then

$$\begin{array}{c} \int_{{\left[0,p\right]}^{d}}C_{d}\left(u\right)du=\int_{{\left[0,1\right]}^{p}}u_{1}u_{2}\cdots u_{d}\left[1+\alpha \left(1-u_{1}\right)\cdots \left(1-u_{d}\right)\right]du={\left(\frac{p^{2}}{2}\right)}^{d}+\alpha {\left(\frac{3p^{2}-2p^{3}}{6}\right)}^{d}. \end{array}$$

Going back to general $C_{d}$, we can notice that the resulting integral would always be a polynomial in *p*, with the lowest power being 2d and thus

$$\begin{array}{cc} \lambda_{L,S}\left(C_{d}\right) & =\lim_{p↘0}\frac{d+1}{p^{d+1}}p^{2d}=0. \end{array}$$

A similar calculation leads to $\lambda_{U,S}\left(C_{d}\right)=0$. Some further calculations (not presented here) also show that $\lambda_{U,S}^{*}\left(C_{d}\right)=0$.

From the perspective of all the above tail dependence coefficients, the Farlie–Gumbel–Morgenstern copula does not possess any tail dependence.

**Example** **5.** *Cuadras-Augé copula.*

Let $C_{d}$ be a *d*-variate Cuadras-Augé copula, that is of the form

$$\begin{array}{c} C_{d}\left(u_{1},\cdots ,u_{d}\right)={\left[\min\left(u_{1},\cdots ,u_{d}\right)\right]}^{\theta }{\left(u_{1}u_{2}\cdots u_{d}\right)}^{1-\theta } \end{array}$$

for θ∈[0,1]. The Cuadras-Augé copula combines the comonotonicity copula $M_{d}$ with the independence copula $\Pi_{d}$. If θ=0, then $C_{d}$ becomes $\Pi_{d}$. If θ=1, then $C_{d}$ becomes $M_{d}$.

We again start with calculating $\epsilon_{L}$ and $\epsilon_{U}$. From (2), we find

C¯d(u1)=1+∑j=1d(−1)jdjuj−(j−1)θ

and Frahm’s lower extremal dependence coefficient $\epsilon_{L}$ is thus given as

ϵL(Cd)=limu↘0Cd(u1)1−C¯d(u1)=limu↘0ud−(d−1)θ∑j=1d(−1)j+1djuj−(j−1)θ=limu↘0ud−(d−1)θ−1∑j=1d(−1)j+1djuj−(j−1)θ−1=1ifθ=1,0ifθ∈[0,1)

since if θ∈[0,1), the polynomial in *u* in the numerator converges to zero faster than the polynomial in the denominator. For $\epsilon_{U}$, using L’Hospital’s rule leads to

ϵU(Cd)=limu↗1C¯d(u1)1−Cd(u1)=limu↗11+∑j=1d(−1)jdjuj−(j−1)θ1−ud−(d−1)θ=limu↗1∑j=1d(−1)jdjj−(j−1)θuj−(j−1)θ−1−(d−(d−1)θ)ud−(d−1)θ−1=∑j=1d(−1)jdjj−(j−1)θ−(d−(d−1)θ)

=(1−θ)∑j=1d(−1)jdjj+θ∑j=1d(−1)jdj−(d−(d−1)θ)=0−θ−(d−(d−1)θ)=θd−(d−1)θ.

These values coincide with those calculated in [20] for a more general group of copulas. One can also notice that

$$\begin{array}{c} \lim_{d\to \infty }\epsilon_{U}\left(C_{d}\right)=\left\{\begin{array}{cc} 1 & \mathrm{if}\theta =1,\\ 0 & \mathrm{if}\theta \in [0,1). \end{array}\right. \end{array}$$

**Figure 3:**
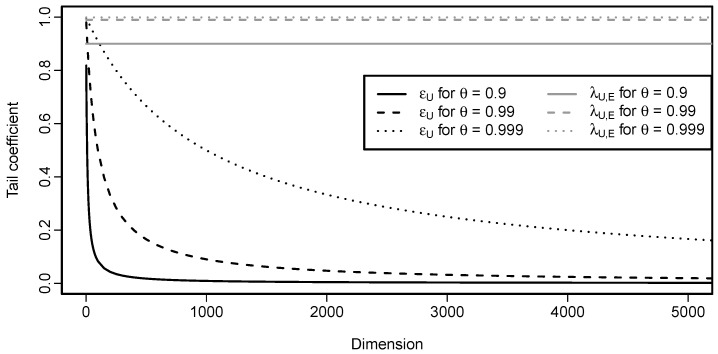
Frahm’s upper extremal dependence coefficient (black line) and tail dependence coefficient for extreme-value copulas $\lambda_{U,E}$ (grey line) for a Cuadras–Augé copula with parameters 0.9 (solid line), 0.99 (dashed line) and 0.999 (dotted line) as a function of the dimension of the copula.

In other words, if the parameter θ is smaller than 1, any sign of tail dependence disappears when the dimension increases. If θ=1, then $\epsilon_{U}\left(C_{d}\right)=1$ for every d≥2 which is no surprise, since, in that case, $C_{d}$ is the comonotonicity copula $M_{d}$. This behavior is illustrated in Figure 3 that details the influence of the parameter θ on the speed of decrease of $\epsilon_{U}\left(C_{d}\right)$ when *d* increases.

A Cuadras–Augé copula is an exchangeable copula, which is invariant with respect to the order of its arguments. Therefore, when calculating Li’s tail dependence parameters, only the cardinality of the index sets $I_{h}$ and $J_{d-h}$ plays a role. Subsequently,

$$\begin{array}{c} \lambda_{L}^{I_{h}|J_{d-h}}\left(C_{d}\right)=\lim_{u↘0}\frac{u^{d-(d-1)\theta }}{u^{d-h-(d-h-1)\theta }}=\left\{\begin{array}{cc} 1 & if\theta =1,\\ 0 & if\theta \in [0,1) \end{array}\right. \end{array}$$

and by using L’Hospital’s rule

(36) λUIh|Jd−h(Cd)=limu↗11+∑j=1d(−1)jdjuj−(j−1)θ1+∑j=1d−h(−1)jd−hjuj−(j−1)θ=∑j=1d(−1)jdj(j−(j−1)θ)∑j=1d−h(−1)jd−hj(j−(j−1)θ).

If θ=1, then $\lambda_{U}^{I_{h}|J_{d-h}}\left(C_{d}\right)=1$, as expected, and it does not depend on the conditioning sets $I_{h}$ and $J_{d-h}$.

For Schmid’s and Schmidt’s lower tail dependence measure $\lambda_{L,S}\left(C_{d}\right)$, defined in (24), we first need to calculate the integral $\int_{{\left[0,p\right]}^{d}}C_{d}\left(u\right)du$. A straightforward calculation gives that

$$\int_{{\left[0,p\right]}^{d}}C_{d}\left(u\right)du=\frac{d}{{\left(2-\theta \right)}^{d}}p^{(2-\theta )(d-1)+2}B\left(\frac{2}{2-\theta },d\right)$$

where $B\left(s,t\right)=\int_{0}^{1}x^{s-1}{\left(1-x\right)}^{t-1}dx$ is the Beta function. We then get

$$\lambda_{L,S}\left(C_{d}\right)=\lim_{p↘0}\frac{d+1}{p^{d+1}}\;\frac{d}{{\left(2-\theta \right)}^{d}}p^{(2-\theta )(d-1)+2}B\left(\frac{2}{2-\theta },d\right),$$

which equals 1 when θ=1 and 0 when θ∈[0,1). Schmid’s and Schmidt’s lower tail dependence measure thus equals Frahm’s lower extremal dependence coefficient $\epsilon_{L}$ as well as Li’s lower tail dependence parameter $\lambda_{L}^{I_{h}|J_{d-h}}\left(C_{d}\right)$.

Determining Schmid’s and Schmidt’s upper tail dependence measure $\lambda_{U,S}\left(C_{d}\right)$ in (25) is less straightforward. This dependence measure involves three integrals. Because its expression concerns the limit when p→0, it suffices to investigate the behavior of the numerator and the denominator of (25) for *p* close to 0. From (27) it is easy to see that, for *p* close to 0,

$$\int_{{\left[1-p,1\right]}^{d}}\Pi_{d}\left(u\right)du=p^{d}-\frac{d}{2}p^{d+1}+o\left(p^{d+1}\right),$$

and, hence, the denominator of (25) behaves, for *p* close to 0, as

(37) $$\int_{{\left[1-p,1\right]}^{d}}M_{d}\left(u\right)du-\int_{{\left[1-p,1\right]}^{d}}\Pi_{d}\left(u\right)du=\frac{d(d-1)}{2(d+1)}p^{d+1}+o\left(p^{d+1}\right).$$

For the integral $\int_{{\left[1-p,1\right]}^{d}}C_{d}\left(u\right)du$, note that, since $C_{d}$ is an exchangeable copula, we can divide the integration domain ${\left[1-p,1\right]}^{d}$ into *d* parts depending on which argument from $u_{1},\cdots ,u_{d}$ is minimal. The integrals over each of the *d* parts are equal. We get

$$\begin{array}{ccc} \int_{{\left[1-p,1\right]}^{d}}C_{d}\left(u\right)du & = & d\int_{1-p}^{1}u_{1}\left(\prod_{j=2}^{d}\int_{u_{1}}^{1}u_{j}^{1-\theta }du_{j}\right)du_{1}\\ & = & d\int_{1-p}^{1}u_{1}{\left(\frac{1-u_{1}^{2-\theta }}{2-\theta }\right)}^{d-1}du_{1}\\ & = & \frac{d}{{\left(2-\theta \right)}^{d-1}}\int_{1-p}^{1}u_{1}{\left(1-u_{1}^{2-\theta }\right)}^{d-1}du_{1}\\ & = & p^{d}+\left[\theta \frac{d(d-1)}{2(d+1)}-\frac{d}{2}\right]p^{d+1}+o\left(p^{d+1}\right), \end{array}$$

where the approximation, valid for *p* close to 0, is based on a careful evaluation of the integral. For brevity, we do not include the details here. Consequently the numerator of (25) behaves, for *p* close to 0, as

(38) $$\int_{{\left[1-p,1\right]}^{d}}C_{d}\left(u\right)du-\int_{{\left[1-p,1\right]}^{d}}\Pi_{d}\left(u\right)du=\theta \frac{d(d-1)}{2(d+1)}p^{d+1}+o\left(p^{d+1}\right).$$

Combining (37) and (38) reveals that $\lambda_{U,S}\left(C_{d}\right)=\theta$, for all d≥2. Other calculations (omitted here for brevity) lead to $\lambda_{U,S}^{*}\left(C_{d}\right)=\theta$.

A Cuadras–Augé copula is also an extreme-value copula. This can be seen through the following calculation, where the notation $u_{\left(1\right)}=\min\left(u_{1},\cdots ,u_{d}\right)$ is used. One gets

$$\begin{array}{cc} C_{d}\left(u_{1},\cdots ,u_{d}\right) & ={\left[u_{\left(1\right)}\right]}^{\theta }{\left(u_{1}u_{2}\cdots u_{d}\right)}^{1-\theta }=\exp\left\{\theta \log\left(u_{\left(1\right)}\right)+\left(1-\theta \right)\sum_{j=1}^{d}\log\left(u_{j}\right)\right\}\\ \; & =\exp\left\{\left(\frac{\theta \log\left(u_{\left(1\right)}\right)}{\log(u_{1}u_{2}\cdots u_{d})}+\frac{\left(1-\theta \right)\sum_{j=1}^{d}\log\left(u_{j}\right)}{\log(u_{1}u_{2}\cdots u_{d})}\right)\log\left(u_{1}u_{2}\cdots u_{d}\right)\right\} \end{array}$$

and, thus, $C_{d}$ is an extreme-value copula with Pickands dependence function

$$\begin{array}{c} A_{d}\left(w_{1},\cdots ,w_{d}\right)=\theta w_{\left(1\right)}+\left(1-\theta \right)\sum_{j=1}^{d}w_{j}. \end{array}$$

This allows for calculating the tail coefficient for extreme-value copulas, $\lambda_{U,E}$, as

$$\begin{array}{c} \lambda_{U,E}\left(C_{d}\right)=\frac{d}{d-1}\left(1-\frac{\theta }{d}-\left(1-\theta \right)\right)=\theta . \end{array}$$

In case of the Cuadras–Augé copula, tail dependence measured by $\lambda_{U,E}$ does not depend on the dimension *d*. For illustration, the values of $\lambda_{U,E}\left(C_{d}\right)$ are included in Figure 3. One can see that $\epsilon_{U}$ and $\lambda_{U,E}$ behave very differently, both in terms of shapes and values.

**Example** **6.** *Clayton copula.*

Let $C_{d}$ be a *d*-variate Clayton family copula defined as

(39) $$\begin{array}{c} C_{d}\left(u\right)={\left(\sum_{j=1}^{d}u_{j}^{-\theta }-d+1\right)}^{-1/\theta } \end{array}$$

for θ>0. The Clayton copula is an Archimedean copula and the behavior of its generator is studied in Example 2.

For Frahm’s lower extremal dependence coefficient, either using (12) or by factoring out as below, one obtains

ϵL(Cd)=limu↘0Cd(u1)1−C¯d(u1)=limu↘0u(d−duθ+uθ)−1/θ∑j=1d(−1)j+1dju(j−juθ+uθ)−1/θ

(40) =d−1/θ∑j=1d(−1)j+1djj−1/θ,

whereas, for Frahm’s upper extremal dependence coefficient, using (13) with the derivative of the Clayton generator $\psi^{\prime }\left(t\right)=-{\left(1+\theta t\right)}^{-(1+\theta )/\theta }$, one finds

ϵU(Cd)=limt↘0∑j=1d(−1)jdjψ′(jt)j−ψ′(dt)d=−∑j=1d(−1)jdjjd=∑j=1d(−1)j+1d−1j−1=0.

**Figure 4:**
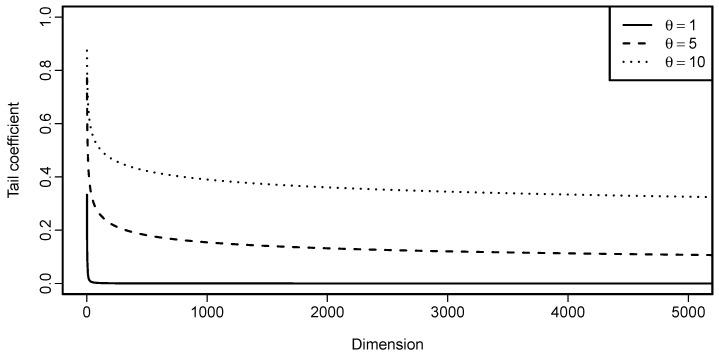
Frahm’s lower extremal dependence coefficient for Clayton copula with parameters 1 (solid line), 5 (dashed line) and 10 (dotted line) as a function of the dimension of the copula.

Analytical calculation of $\lim_{d\to \infty }\epsilon_{L}\left(C_{d}\right)$ is not possible; however, insight can be gained by plotting $\epsilon_{L}\left(C_{d}\right)$ as a function of the dimension *d*. This is done in Figure 4. From the plot it is evident that $\epsilon_{L}\left(C_{d}\right)$ decreases when the dimension increases. However, for larger parameter values, the decrease seems to be slow.

A Clayton copula is also an exchangeable copula and, thus, when calculating Li’s tail dependence parameters, only the cardinality of the index sets $I_{h}$ and $J_{d-h}$ comes into play. Then

(41) $$\begin{array}{cc} \lambda_{L}^{I_{h}|J_{d-h}}\left(C_{d}\right) & =\lim_{u↘0}\frac{{\left(du^{-\theta }-d+1\right)}^{-1/\theta }}{{\left(\left(d-h\right)u^{-\theta }-\left(d-h\right)+1\right)}^{-1/\theta }}=\lim_{u↘0}\frac{{\left(d-du^{\theta }+u^{\theta }\right)}^{-1/\theta }}{{\left(d-h-\left(d-h\right)u^{\theta }+u^{\theta }\right)}^{-1/\theta }}\\ \; & ={\left(\frac{d-h}{d}\right)}^{1/\theta }. \end{array}$$

If, as in Proposition 11, the cardinality of $J_{d-h}$ is kept constant (equal to $h^{*}$) when the dimension increases, then

(42) $$\begin{array}{c} \lim_{d\to \infty }\lambda_{L}^{I_{h}|J_{d-h}}\left(C_{d}\right)=0. \end{array}$$

In fact, in this example, even a milder condition is sufficient for achieving (42). If h=h(d) is linked to the dimension such that $\lim_{d\to \infty }\left(d-h\left(d\right)\right)/d=0$, then (42) holds. However, for large values of the parameter θ, the convergence in (42) might be very slow. By applying L’Hospital’s rule (d−h) times, one can also calculate

$$\begin{array}{cc} \lambda_{U}^{I_{h}|J_{d-h}}\left(C_{d}\right) & =0. \end{array}$$

Spearman’s rho for the Clayton copula cannot be explicitly calculated and, thus, the values of $\lambda_{L,S}$ and $\lambda_{U,S}$ are unknown.

**Example** **7.** *Gumbel-Hougaard copula.*

Let $C_{d}$ be a *d*-variate Gumbel–Hougaard copula, defined as

$$\begin{array}{c} C_{d}\left(u\right)=\exp\left\{-{\left[\sum_{j=1}^{d}{\left(-\log u_{j}\right)}^{\theta }\right]}^{1/\theta }\right\} \end{array}$$

where θ≥1. The Gumbel-Hougaard copula is the only copula (family) that is both an extreme-value and an Archimedean copula as proved in [21] (Sec. 2). The behavior of its Archimedean generator is studied in Example 3. Note that θ=1 corresponds to the independence copula $\Pi_{d}$ and the limiting case θ→∞ corresponds to the comonotonicity copula $M_{d}$.

As expected (see (10)), for an extreme-value copula which is not the comonotonicity copula, the Frahm’s lower extremal dependence coefficient is

ϵL(Cd)=limu↘0Cd(u1)1−C¯d(u1)=limu↘0ud1/θ∑j=1d(−1)j+1djuj1/θ=0

since the polynomial in *u* in the numerator converges to zero faster than the polynomial in the denominator. For the Frahm’s upper extremal dependence coefficient, by using (13) with the derivative of the Gumbel–Hougaard generator $\psi^{\prime }\left(t\right)=\frac{-1}{\theta }\exp\left(-t^{1/\theta }\right)t^{1/\theta -1}$, one obtains

(43) ϵU(Cd)=limt↘0∑j=1d(−1)jdjψ′(jt)j−ψ′(dt)d=limt↘0−1θt1/θ−1∑j=1d(−1)jdjexp(−(jt)1/θ)j1/θ1θt1/θ−1exp(−(dt)1/θ)d1/θ=∑j=1d(−1)j+1djj1/θd1/θ.

**Figure 5:**
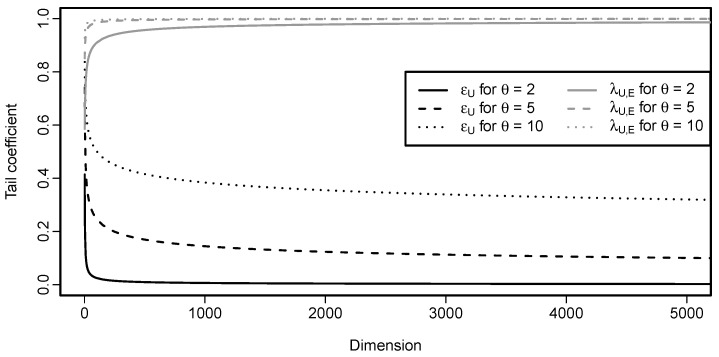
Frahm’s upper extremal dependence coefficient (black line) and tail dependence coefficient for extreme-value copulas $\lambda_{U,E}$ (grey line) for Gumbel–Hougaard copula with parameters 2 (solid line), 5 (dashed line) and 10 (dotted line) as a function of the dimension of the copula.

Analytical calculation of $\lim_{d\to \infty }\epsilon_{U}\left(C_{d}\right)$ is not possible; however, insights can be gained by plotting $\epsilon_{U}\left(C_{d}\right)$ as a function of dimension *d*. This is done in Figure 5. It is evident that $\epsilon_{U}\left(C_{d}\right)$ decreases when the dimension increases; but, the decrease seems to be slow for larger parameter values. When comparing Figure 4 and Figure 5, one might come to a conclusion that $\epsilon_{L}$ for the Clayton copula with parameter θ is equal to $\epsilon_{U}$ for the Gumbel–Hougaard copula with the same parameter θ. Despite their similarity, that is not true, as can be easily checked by calculating both of the quantities for any pair (d,θ).

When calculating Li’s tail dependence parameters, one uses that the Gumbel–Hougaard copula is also an exchangeable copula and, thus, only the cardinality of the index sets $I_{h}$ and $J_{d-h}$ plays a role. Then

$$\begin{array}{cc} \lambda_{L}^{I_{h}|J_{d-h}}\left(C_{d}\right) & =\lim_{u↘0}\frac{u^{d^{1/\theta }}}{u^{{\left(d-h\right)}^{1/\theta }}}=0. \end{array}$$

If θ=1, then $\lambda_{U}^{I_{h}|J_{d-h}}\left(C_{d}\right)=0$, otherwise by using L’Hospital’s rule

(44) λUIh|Jd−h(Cd)=limu↗1∑j=0d(−1)jdjuj1/θ∑j=0d−h(−1)jd−hjuj1/θ=∑j=1d(−1)jdjj1/θ∑j=1d−h(−1)jd−hjj1/θ.

**Figure 6:**
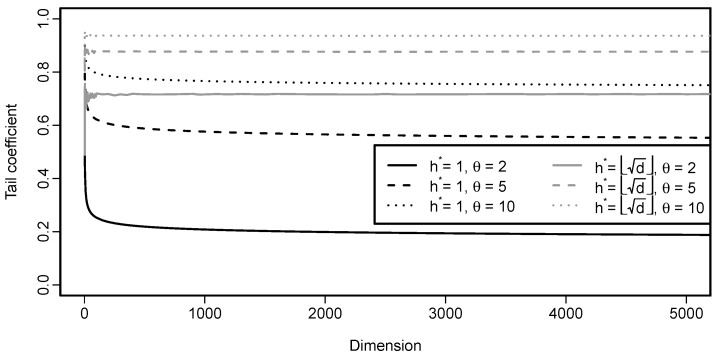
Li’s upper tail dependence parameter with $h^{*}=1$ (black line) and with $h^{*}=\left\lfloor \sqrt{d}\right\rfloor$ (grey line) for Gumbel-Hougaard copula with parameters 2 (solid line), 5 (dashed line) and 10 (dotted line) as a function of the dimension of the copula.

This function of parameter θ, dimension *d* and cardinality *h* is rather involved and it is depicted in Figure 6 for different parameter choices and also two different selections of *h*. In one of the cases, h=d−1 and thus corresponds to $h^{*}=1$ in Proposition 11. In the other case, the number of components on which we condition $h^{*}=h^{*}\left(d\right)$ is chosen to increase with *d*, specifically $h^{*}\left(d\right)=\left\lfloor \sqrt{d}\right\rfloor$. For $h^{*}=1$ (and thus the setting of Proposition 11), the tail coefficient slowly decreases with dimension, as expected. An interesting behavior is seen for $h^{*}\left(d\right)=\left\lfloor \sqrt{d}\right\rfloor$, where the tail coefficient seems to be, except for instability in low dimensions, constant, independently of the parameter θ choice.

Spearman’s rho for a Gumbel–Hougaard copula cannot be calculated explicitly and thus the values of $\lambda_{L,S}$ and $\lambda_{U,S}$ are unknown.

Pickands dependence function $A_{d}$ of a Gumbel–Hougaard copula is

$$\begin{array}{c} A_{d}\left(w\right)={\left(w_{1}+\cdots +w_{d}\right)}^{-1}{\left(w_{1}^{\theta }+\cdots +w_{d}^{\theta }\right)}^{1/\theta } \end{array}$$

and thus

$$\begin{array}{c} \lambda_{U,E}\left(C_{d}\right)=\frac{d-d^{1/\theta }}{d-1}. \end{array}$$

Note that $\lim_{d\to \infty }\lambda_{U,E}\left(C_{d}\right)=1$. From our perspective, such a behavior is rather counter-intuitive and should be taken into account when using this tail coefficient.

An overview of the results obtained in the illustrative examples is given in Table 2.

## 7. Estimation of Tail Coefficients

Before we move to the estimation of tail coefficients itself, we introduce the setting and notation for the estimation.

### 7.1. Preliminaries

Let $X_{1},\cdots ,X_{n}$ be a random sample of a *d*-dimensional random vector with copula $C_{d}$ where $X_{i}={\left(X_{1,i},\cdots X_{d,i}\right)}^{⊤}$ for i∈{1,⋯n}. Throughout this section, the dimension *d* of a copula $C_{d}$ is arbitrary but fixed and, thus, for simplicity of notation, we omit the subscript *d* in $C_{d}$.

We consider the empirical copula

(45) $${\hat{C}}_{n}\left(u\right)=\frac{1}{n}\sum_{i=1}^{n}𝟙\left({\hat{U}}_{1,i}\leq u_{1},\cdots ,{\hat{U}}_{d,i}\leq u_{d}\right),$$

where

$${\hat{U}}_{j,i}={\hat{F}}_{j,n}\left(X_{j,i}\right),\;\mathrm{with}\;{\hat{F}}_{j,n}\left(x\right)=\frac{1}{n+1}\sum_{i=1}^{n}𝟙\left(X_{j,i}\leq x\right),\;x\in R.$$

Similarly, we define the empirical survival function as

$${\hat{\bar{C}}}_{n}\left(u\right)=\frac{1}{n}\sum_{i=1}^{n}𝟙\left({\hat{U}}_{1,i}>u_{1},\cdots ,{\hat{U}}_{d,i}>u_{d}\right).$$

For extreme-value copulas, one can take advantage of estimation methods for the Pickands dependence function or the stable tail dependence function. The estimation of these was discussed, for example, in [22,23,24], or [7]. We briefly discuss the estimator for the Pickands dependence function, as proposed in [7].

#### Madogram Estimator of Pickands Dependence Function

The multivariate w-madogram, as introduced in [7], is, for $w\in \Delta_{d-1}$, defined as

$$\begin{array}{c} \nu_{d}\left(w\right)=E\left(\overset{d}{\underset{j=1}{⋁}}F_{j}^{1/w_{j}}\left(X_{j}\right)-\frac{1}{d}\sum_{j=1}^{d}F_{j}^{1/w_{j}}\left(X_{j}\right)\right), \end{array}$$

where $u^{1/w_{j}}=0$ by convention if $w_{j}=0$ and 0<u<1. The authors in [7] further show a relation between Pickands dependence function and the madogram given by

$$\begin{array}{c} A_{d}\left(w\right)=\frac{\nu_{d}\left(w\right)+c\left(w\right)}{1-\nu_{d}\left(w\right)-c\left(w\right)} \end{array}$$

where $c\left(w\right)=d^{-1}\sum_{j=1}^{d}w_{j}/\left(1+w_{j}\right)$. This leads to the following estimator of Pickands dependence function

$$\begin{array}{cc} {\hat{A}}_{n}^{\mathrm{MD}}\left(w\right) & =\frac{{\hat{\nu }}_{n}\left(w\right)+c\left(w\right)}{1-{\hat{\nu }}_{n}\left(w\right)-c\left(w\right)} \end{array}$$

with

$$\begin{array}{cc} {\hat{\nu }}_{n}\left(w\right) & =\frac{1}{n}\sum_{i=1}^{n}\left(\overset{d}{\underset{j=1}{⋁}}{\hat{F}}_{j,n}^{1/w_{j}}\left(X_{j,i}\right)-\frac{1}{d}\sum_{j=1}^{d}{\hat{F}}_{j,n}^{1/w_{j}}\left(X_{j,i}\right)\right). \end{array}$$

However, the estimator ${\hat{A}}_{n}^{\mathrm{MD}}$ is not a proper Pickands dependence function. To deal with this problem, they propose an estimator based on Bernstein polynomials that overcomes this issue and results into an estimator, which is a proper Pickands dependence function.

### 7.2. Estimation of the Various Tail Coefficients

#### 7.2.1. Estimation of Frahm’s Extremal Dependence Coefficient

The estimation of the Frahm’s extremal dependence coefficients has not been discussed in the literature so far. However, a straightforward approach is to consider empirical approximations of the quantities in definition (7), i.e.,

$${\hat{\epsilon }}_{L}=\frac{{\hat{C}}_{n}\left(u_{n},\cdots ,u_{n}\right)}{1-{\hat{\bar{C}}}_{n}\left(u_{n},\cdots ,u_{n}\right)},\;{\hat{\epsilon }}_{U}=\frac{{\hat{\bar{C}}}_{n}\left(1-u_{n},\cdots ,1-u_{n}\right)}{1-{\hat{C}}_{n}\left(1-u_{n},\cdots ,1-u_{n}\right)},$$

where $\left\{u_{n}\right\}$ is a sequence of positive numbers converging to zero. The choice of $u_{n}$ is crucial for the performance of the estimator. Small values of $u_{n}$ provide an estimator with low bias but large variance, large values of $u_{n}$ provide an estimator with large bias but small variance. Note that, in applications, it is useful to think about $u_{n}$ as $u_{n}=\frac{k_{n}}{n+1}$, where $k_{n}$ stands for the numbers of extreme values used in the estimation procedure.

Alternatively, if the underlying copula is known to be an extreme-value copula, the estimator can be based on the estimator of Pickands dependence function plugged into (11). This results in the following estimator

$$\begin{array}{c} {\hat{\epsilon }}_{U}^{\mathrm{MD}}=\frac{\sum_{j=1}^{d}{\left(-1\right)}^{j+1}\sum_{1\leq k_{1}<\cdots <k_{j}\leq d}\;j\;{\hat{A}}_{n}^{MD}\left(w_{1},\cdots ,w_{d}\right)}{d{\hat{A}}_{n}^{MD}\left(1/d,\cdots ,1/d\right)}, \end{array}$$

with $w_{\ell }=1/j$ if $\ell \in \{k_{1},\cdots ,k_{j}\}$ and $w_{\ell }=0$ otherwise.

#### 7.2.2. Estimation of Li’s Tail Dependence Parameters

Similarly as for Frahm’s extremal dependence coefficients, one can introduce the following estimators

$$\begin{array}{c} {\hat{\lambda }}_{L}^{I_{h}|J_{d-h}}=\frac{{\hat{C}}_{n}\left(u_{n},\cdots ,u_{n}\right)}{{\hat{C}}_{n}^{J_{d-h}}\left(u_{n},\cdots ,u_{n}\right)},\;{\hat{\lambda }}_{U}^{I_{h}|J_{d-h}}=\frac{{\hat{\bar{C}}}_{n}\left(1-u_{n},\cdots ,1-u_{n}\right)}{{\hat{\bar{C}}}_{n}^{J_{d-h}}\left(1-u_{n},\cdots ,1-u_{n}\right)}. \end{array}$$

#### 7.2.3. Estimation of Schmid’s and Schmidt’s Tail Dependence Measure

Also in this case, one can make use of the empirical copula (45). Recall the definition of $\lambda_{L,S}$ in (24), and consider *p* small. More precisely, let $p_{n}$ be a small positive number. Subsequently, one can calculate

(46) $$\begin{array}{c} \int_{{\left[0,p_{n}\right]}^{d}}{\hat{C}}_{n}\left(u\right)du=\frac{1}{n}\sum_{i=1}^{n}\prod_{j=1}^{d}{\left(p_{n}-{\hat{U}}_{j,i}\right)}_{+}. \end{array}$$

The estimator of $\lambda_{L,S}$ that could then be considered is of the form

$$\begin{array}{c} \frac{\left(d+1\right)}{n\;p_{n}^{d+1}}\sum_{i=1}^{n}\prod_{j=1}^{d}{\left(p_{n}-{\hat{U}}_{j,i}\right)}_{+}. \end{array}$$

However, this quantity does not provide the value 1 for a sample from a comonotonicity copula. See the related discussion in [25]. This problem increases, while $p_{n}$ gets smaller. Thus, we propose to use an estimator defined as

$$\begin{array}{c} {\hat{\lambda }}_{L,S}=\frac{\sum_{i=1}^{n}\prod_{j=1}^{d}{\left(p_{n}-{\hat{U}}_{j,i}\right)}_{+}}{\sum_{i=1}^{n}{\left[{\left(p_{n}-\frac{i}{n+1}\right)}_{+}\right]}^{d}} \end{array}$$

where the denominator is based on estimating $\int_{{\left[0,p\right]}^{d}}M_{d}\left(u\right)u$ using (46) and the fact that for a sample from a comonotonicity copula ${\hat{U}}_{1,i}=\cdots ={\hat{U}}_{d,i}$ for every i∈{1,⋯,n} almost surely. Analogous arguments lead to an estimator of $\lambda_{U,S}^{*}$, as defined in (26), given by

$$\begin{array}{c} {\hat{\lambda }}_{U,S}^{*}=\frac{\sum_{i=1}^{n}\prod_{j=1}^{d}{\left(p_{n}-\left(1-{\hat{U}}_{j,i}\right)\right)}_{+}}{\sum_{i=1}^{n}{\left[{\left(p_{n}-\frac{i}{n+1}\right)}_{+}\right]}^{d}}. \end{array}$$

#### 7.2.4. Estimation of $\lambda_{U,E}$ the Proposed Tail Coefficient for Extreme-Value Copulas

Because coefficient $\lambda_{U,E}$, in (28), is a function of Pickands dependence function $A_{d}$, estimation can again be based on estimation of $A_{d}$. For example, the madogram estimator ${\hat{A}}_{n}^{\mathrm{MD}}$ can be used, which results in the following estimator

$$\begin{array}{c} {\hat{\lambda }}_{U,E}^{\mathrm{MD}}=\frac{d}{d-1}\left(1-{\hat{A}}_{n}^{\mathrm{MD}}\left(1/d,\cdots ,1/d\right)\right). \end{array}$$

The consistency results for the suggested estimators can be found in the following propositions.

**Proposition** **12.** *Suppose that $C_{d}$ is a d-variate extreme-value copula. Subsequently, the estimators ${\hat{\epsilon }}_{U}^{\mathrm{MD}}$ and ${\hat{\lambda }}_{U,E}^{\mathrm{MD}}$ are strongly consistent.*

**Proof.**  The statement of the proposition follows by Theorem 2.4(b) in [7], which states that

$$\underset{w\in \Delta_{d-1}}{\sup}|{\hat{A}}_{n}^{\mathrm{MD}}\left(w\right)-A\left(w\right)|\overset{\mathrm{alm}.\;\mathrm{surely}}{\underset{n\to \infty }{\to }}0.$$

 □

**Proposition** **13.** *Suppose that $u_{n},p_{n}\in \left(n^{-\delta },n^{-\gamma }\right)$ for some 0<γ<δ<1.*

*(i)*  *Then ${\hat{\epsilon }}_{L}$ and ${\hat{\epsilon }}_{U}$ are weakly consistent.*
*(ii)*  *Then ${\hat{\lambda }}_{L,S}$ and ${\hat{\lambda }}_{U,S}^{*}$ are weakly consistent.*
*(iii)*  *Further suppose that $(n\;C^{J_{d-h}}\left(u_{n}1\right))\to \infty$. Subsequently, the following implications hold.*
*If $\;\lim_{\gamma \to 0}\lim_{u\to 0_{+}}\frac{C^{J_{d-h}}\left(u\left(1+\gamma \right)1\right)}{C^{J_{d-h}}\left(u1\right)}=1$, then ${\hat{\lambda }}_{L}^{I_{h}|J_{d-h}}$ is weakly consistent.*
*If $\;\lim_{\gamma \to 0}\lim_{u\to 0_{+}}\frac{{\bar{C}}^{J_{d-h}}\left(u\left(1+\gamma \right)1\right)}{{\bar{C}}^{J_{d-h}}\left(u1\right)}=1$, then ${\hat{\lambda }}_{U}^{I_{h}|J_{d-h}}$ is weakly consistent.*

**Proof.**  We will only deal with the estimators of the lower dependence coefficients ${\hat{\epsilon }}_{L}$, ${\hat{\lambda }}_{L,S}$ and ${\hat{\lambda }}_{L}^{I_{h}|J_{d-h}}$. The estimators of the upper dependence coefficients can be handled completely analogously. *Showing (i)*.

With the help of (A.22) of [26], one gets that for each $\beta <\frac{1}{2}$

$${\hat{U}}_{j,i}=U_{j,i}+U_{j,i}^{\beta }\;O_{P}\left(\frac{1}{\sqrt{n}}\right),\;\mathrm{uniformly}\;\mathrm{in}\;j\in \left\{1,\cdots ,d\right\},i\in \left\{1,\cdots ,n\right\}.$$

This, together with Lemma A3 in [27] (see also (A.12) in [26]), implies that, for each ε>0 with probability arbitrarily close to 1 for all sufficiently large *n*, it holds that

(47) $$\left[U_{j,i}\leq u_{n}\left(1-\varepsilon \right)\right]\subseteq \left[{\hat{U}}_{j,i}\leq u_{n}\right]\subseteq \left[U_{j,i}\leq u_{n}\left(1+\varepsilon \right)\right],\;\mathrm{for}\;\mathrm{all}\;j,i.$$

Denote

$$G_{n}\left(u\right)=\frac{1}{n}\sum_{i=1}^{n}𝟙\left\{U_{i}\leq u\right\}.$$

Subsequently, conditionally on (47) and with the help of Chebyshev’s inequality, one gets that

(48) $$\begin{array}{cc} {\hat{C}}_{n}\left(u_{n}1\right)\; & \leq G_{n}\left(u_{n}\left(1+\varepsilon \right)1\right)=C\left(u_{n}\left(1+\varepsilon \right)1\right)+\sqrt{C\left(u_{n}\left(1+\varepsilon \right)1\right)}\;O_{P}\left(\frac{1}{\sqrt{n}}\right) \end{array}$$

(49) $$\begin{array}{cc} \; & =C\left(u_{n}1\right)+\varepsilon \;O\left(u_{n}\right)+\sqrt{u_{n}}\;O_{P}\left(\frac{1}{\sqrt{n}}\right). \end{array}$$

Analogously, also

(50) $${\hat{C}}_{n}\left(u_{n}1\right)\geq C\left(u_{n}1\right)+\varepsilon \;O\left(u_{n}\right)+\sqrt{u_{n}}\;O_{P}\left(\frac{1}{\sqrt{n}}\right).$$

As ε>0 is arbitrary, one can combine (49) and (50) to deduce that

(51) $${\hat{C}}_{n}\left(u_{n}1\right)=C\left(u_{n}1\right)+o_{P}\left(u_{n}\right).$$

Completely analogously with the help of (2), one can show that

(52) $$1-{\hat{\bar{C}}}_{n}\left(u_{n}1\right)=1-\bar{C}\left(u_{n}1\right)+o_{P}\left(u_{n}\right).$$

Further note that

(53) $$1-\bar{C}\left(u_{n}1\right)=P\left(U_{\min}\leq u_{n}\right)\geq P\left(U_{1}\leq u_{n}\right)=u_{n}.$$

Now combining (51), (52) and (53) yields that

$${\hat{\epsilon }}_{L}=\frac{{\hat{C}}_{n}\left(u_{n}1\right)}{1-{\hat{\bar{C}}}_{n}\left(u_{n}1\right)}=\frac{C\left(u_{n}1\right)+o_{P}\left(u_{n}\right)}{1-\bar{C}\left(u_{n}1\right)+o_{P}\left(u_{n}\right)}=\frac{C(u_{n}1)}{1-\bar{C}\left(u_{n}1\right)}+o_{P}\left(1\right)\overset{P}{\underset{n\to \infty }{\to }}\epsilon_{L}.$$

*Showing (ii).*

First of all, note that it is sufficient to show that

(54) $$I_{n}=\frac{d+1}{p_{n}^{d+1}}\int_{{\left[0,p_{n}\right]}^{d}}\left[{\hat{C}}_{n}\left(u\right)-C\left(u\right)\right]du=o_{P}\left(1\right).$$

Further, it is straightforward to bound

(55) $$\begin{array}{cc} \frac{d+1}{p_{n}^{d+1}} & \int_{{\left[0,p_{n}\right]}^{d}\backslash {\left[\frac{p_{n}}{\log n},p_{n}\right]}^{d}}\left|{\hat{C}}_{n}\left(u\right)-C\left(u\right)\right|du\leq \frac{d+1}{p_{n}^{d+1}}\int_{{\left[0,p_{n}\right]}^{d}\backslash {\left[\frac{p_{n}}{\log n},p_{n}\right]}^{d}}\left\{2\min\left\{u_{1},\cdots ,u_{d}\right\}+\frac{1}{n}\right\}\;du\\ \; & \leq \frac{2d(d+1)}{p_{n}^{d+1}}\int_{0}^{p_{n}}\cdots \int_{0}^{p_{n}}\left[\int_{0}^{\frac{p_{n}}{\log n}}u_{1}du_{1}\right]du_{2}\cdots du_{d}+O\left(\frac{1}{n\;p_{n}}\right)=O\left(\frac{1}{\log^{2}n}\right)=o\left(1\right). \end{array}$$

Now, (47) holds uniformly for $u_{n}\in \left[\frac{p_{n}}{\log n},p_{n}\right]$. Thus analogously as one derived (51) one can also show that uniformly in $u\in {\left[\frac{p_{n}}{\log n},p_{n}\right]}^{d}$

$${\hat{C}}_{n}\left(u\right)=C\left(u\right)+o_{P}\left(\sum_{j=1}^{d}u_{j}\right),$$

which further implies

(56) $$\frac{d+1}{p_{n}^{d+1}}\int_{{\left[\frac{p_{n}}{\log n},p_{n}\right]}^{d}}|{\hat{C}}_{n}\left(u\right)-C\left(u\right)|du=o_{P}\left(1\right).$$

Now, combining (55) and (56) yields (54).

*Showing (iii).*

To prove the weak consistency of ${\hat{\lambda }}_{L}^{I_{h}|J_{d-h}}$, it is sufficient to show that

(57) $$\frac{{\hat{C}}_{n}\left(u_{n}1\right)-C\left(u_{n}1\right)}{C^{J_{d-h}}\left(u_{n}1\right)}\overset{P}{\underset{n\to \infty }{\to }}1\;\mathrm{and}\;\frac{{\hat{C}}_{n}^{J_{d-h}}\left(u_{n}1\right)}{C^{J_{d-h}}\left(u_{n}1\right)}\overset{P}{\underset{n\to \infty }{\to }}1.$$

We start with the second convergence. Similarly, as in (48) for each ε>0 with probability arbitrarily close to 1 for all sufficiently large *n*, one can bound

$$\frac{G_{n}^{J_{d-h}}\left(u_{n}\left(1-\varepsilon \right)1\right)}{C^{J_{d-h}}\left(u_{n}\left(1-\varepsilon \right)1\right)}\;\frac{C^{J_{d-h}}\left(u_{n}\left(1-\varepsilon \right)1\right)}{C^{J_{d-h}}\left(u_{n}1\right)}\leq \frac{{\hat{C}}_{n}^{J_{d-h}}\left(u_{n}1\right)}{C^{J_{d-h}}\left(u_{n}1\right)}\leq \frac{G_{n}^{J_{d-h}}\left(u_{n}\left(1+\varepsilon \right)1\right)}{C^{J_{d-h}}\left(u_{n}\left(1+\varepsilon \right)1\right)}\;\frac{C^{J_{d-h}}\left(u_{n}\left(1+\varepsilon \right)1\right)}{C^{J_{d-h}}\left(u_{n}1\right)}\;.$$

Now, by the assumption in (iii), the ratios $\frac{C^{J_{d-h}}\left(u_{n}\left(1-\varepsilon \right)1\right)}{C^{J_{d-h}}\left(u_{n}1\right)}$ and $\frac{C^{J_{d-h}}\left(u_{n}\left(1+\varepsilon \right)1\right)}{C^{J_{d-h}}\left(u_{n}1\right)}$ can be made arbitrarily close to 1 for ε close enough to zero and *n* large enough. Further, by Chebyshev’s inequality

$$\frac{G_{n}^{J_{d-h}}\left(u_{n}\left(1+\varepsilon \right)1\right)}{C^{J_{d-h}}\left(u_{n}\left(1+\varepsilon \right)1\right)}=1+O_{P}\left(\frac{1}{\sqrt{n\;C^{J_{d-h}}\left(u_{n}\left(1+\varepsilon \right)1\right)}}\right)\overset{P}{\underset{n\to \infty }{\to }}1$$

and, similarly, one can show also $\frac{G_{n}^{J_{d-h}}\left(u_{n}\left(1-\varepsilon \right)1\right)}{C^{J_{d-h}}\left(u_{n}\left(1-\varepsilon \right)1\right)}\overset{P}{\underset{n\to \infty }{\to }}1.$ This concludes the proof of the second convergence in (57).

To show the first convergence in (57), one can proceed as in (48) (exploiting (47)) and arrive at

$$\begin{array}{cc} \frac{{\hat{C}}_{n}\left(u_{n}1\right)-C\left(u_{n}1\right)}{C^{J_{d-h}}\left(u_{n}1\right)}\; & \leq \frac{G_{n}\left(u_{n}\left(1+\varepsilon \right)1\right)-C\left(u_{n}\left(1+\varepsilon \right)1\right)}{C^{J_{d-h}}\left(u_{n}1\right)}+\frac{C\left(u_{n}\left(1+\varepsilon \right)1\right)-C\left(u_{n}1\right)}{C^{J_{d-h}}\left(u_{n}1\right)}\\ \; & =O_{P}\left(\frac{1}{\sqrt{n\;C^{J_{d-h}}\left(u_{n}1\right)}}\right)+\frac{C\left(u_{n}\left(1+\varepsilon \right)1\right)-C\left(u_{n}1\right)}{C^{J_{d-h}}\left(u_{n}1\right)}. \end{array}$$

Now, the second term on the right-hand side of the last inequality can be rewritten as

$$\frac{C\left(u_{n}\left(1+\varepsilon \right)1\right)-C\left(u_{n}1\right)}{C^{J_{d-h}}\left(u_{n}1\right)}=\frac{C(u_{n}\left(1+\varepsilon \right)1)}{C^{J_{d-h}}\left(u_{n}\left(1+\varepsilon \right)1\right)}\frac{C^{J_{d-h}}\left(u_{n}\left(1+\varepsilon \right)1\right)}{C^{J_{d-h}}\left(u_{n}1\right)}-\frac{C(u_{n}1)}{C^{J_{d-h}}\left(u_{n}1\right)},$$

which, thanks to the assumptions of the theorem and the existence of $\lambda_{L}^{I_{h}|I_{d-h}}$, can be made arbitrarily small by taking ε small enough and *n* sufficiently large.

As an analogous lower bound can be derived for $\frac{{\hat{C}}_{n}\left(u_{n}1\right)-C\left(u_{n}1\right)}{C^{J_{d-h}}\left(u_{n}1\right)}$, one can conclude that the first convergence in (57) also holds. □

## 8. Real Data Application

In this section, we illustrate the practical use of the multivariate tail coefficients via a real data example. The data concern stock prices of companies that are constituents of the EURO STOXX 50 market index. EURO STOXX 50 index is based on the largest and the most liquid stocks in the eurozone. Daily adjusted prices of these stocks are publicly available on https://finance.yahoo.com/ (downloaded 19 March 2020). The selected time period is 15 years, starting on 18 March 2005 and ending on 18 March 2020. Note that this period covers both the global financial crisis 2007–2008, as well as the sharp decline of the markets that was caused by COVID-19 coronavirus pandemic in early 2020. All the calculations are done in the statistical software R [28]. The R codes for the data application, written by the authors, are available at https://www.karlin.mff.cuni.cz/~omelka/codes.php.

The preprocessing of the data was done, as follows. The stocks are traded on different stock exchanges and thus might differ in trading days. The union of all trading days is used and missing data introduced by this method are filled in by linear interpolation. No data were missing on the first or the last day of the studied time range. Negative log-returns are calculated from the adjusted stock prices and ARMA(1,1)–GARCH(1,1) is fitted to each of the variables (stocks), similarly as for example in [29]. We also refer therein for detailed model specification. Fitting ARMA(1,1)–GARCH(1,1) model to every stock does not necessarily provide the best achievable model, but residual checks show that the models are adequate. The standardized residuals obtained from these univariate models are used as the final dataset for calculating various tail coefficients. The total number of observations is n=3847. Table 3 summarizes the stocks used for the analysis.

It is of interest here to discuss tendency of extremely low returns happening simultaneously, which translates into calculating upper tail coefficients while working with negative log-returns. This allows us to use also the methods assuming that the data are coming from an extreme-value copula.

Six different settings are considered: stocks from Group 1 (G1), from Group 2 (G2), from Group 3 (G3), from G1 and G2, from G1 and G3, and finally stocks from G2 and G3. The dimension *d* is equal to 3 for the first three settings and equal to 6 for the last three settings.

Six different estimators are considered: ${\hat{\epsilon }}_{U}$, ${\hat{\epsilon }}_{U}^{\mathrm{MD}}$, ${\hat{\lambda }}_{U,S}^{*}$, ${\hat{\lambda }}_{U,E}$, and ${\hat{\lambda }}_{U}^{I_{h}|J_{d-h}}$ with two different selections of the conditioning sets $I_{h}$ and $J_{d-h}$. In one case, $h^{*}=d-h=1$ and we condition on only one variable. The specific choice of that one variable does not impact the result, as follows from (19). The analysis with the conditioning on only one variable shows how the rest of the group is affected by the behavior of one stock. In the other case, we condition on all of the stocks, except for the one with largest market capitalization within the group. This analysis indicates how the largest player is affected by the behavior of the rest of the group.

**Figure 7:**
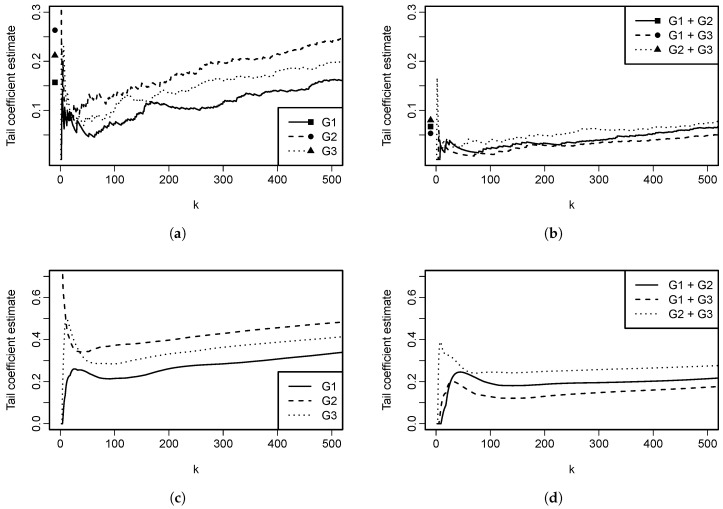
Various estimated tail coefficients. (**a**) Estimator ${\hat{\epsilon }}_{U}$ for 3-variate groups. Corresponding symbols (■, •, ▲) represent values of ${\hat{\epsilon }}_{U}^{\mathrm{MD}}$ (not a function of *k*); (**b**) Estimator ${\hat{\epsilon }}_{U}$ for 6-variate groups. Corresponding symbols (■, •, ▲) represent values of ${\hat{\epsilon }}_{U}^{\mathrm{MD}}$ (not a function of *k*); (**c**) Estimator ${\hat{\lambda }}_{U,S}^{*}$ for 3-variate groups; (**d**) Estimator ${\hat{\lambda }}_{U,S}^{*}$ for 6-variate groups.

**Figure 8:**
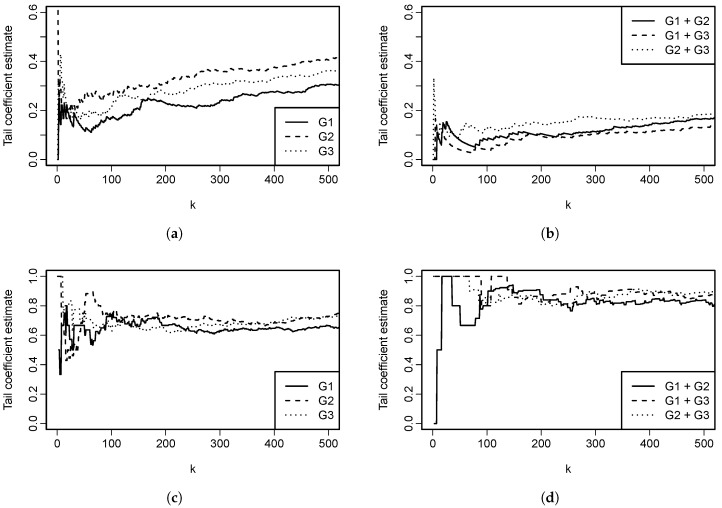
Various estimated tail coefficients. (**a**) Estimator ${\hat{\lambda }}_{U}^{I_{2}|J_{1}}$ for 3-variate groups with conditioning on one stock; (**b**) Estimator ${\hat{\lambda }}_{U}^{I_{5}|J_{1}}$ for 6-variate groups with conditioning on one stock; (**c**) Estimator ${\hat{\lambda }}_{U}^{I_{1}|J_{2}}$ for 3-variate groups with conditioning on all but the stock with highest market capitalization; (**d**) Estimator ${\hat{\lambda }}_{U}^{I_{1}|J_{5}}$ for 6-variate groups with conditioning on all but the stock with highest market capitalization.

The estimators that are functions of the amount of data points *k* (recall from Section 7.2 that a common choice is $u_{n}=k_{n}/\left(n+1\right)$, with $k_{n}=k$ here) do not provide one specific estimate but rather a function of *k*. A selection of in some sense the best possible *k* requires further study. Intuitively, one should look at lowest *k* for which the estimator is not too volatile. This idea was used in [30] for estimating bivariate tail coefficients by finding a plateau in the considered estimator as a function of *k*. The results of the analysis are summarized in Figure 7 and Figure 8 and Table 4. Examining Figure 7, it seems that *k* around 100 would be a possible reasonable choice for the tail coefficients of Frahm, and Schmid and Schmidt, for these data. For Li’s tail dependence parameters, it appears from Figure 8 that, when conditioning on more than one variable, a larger value for *k* is needed, for example k=200.

For the tail dependence measurements for extreme-value copulas, we include the coefficients $\lambda_{U,E}$ and the original extremal coefficient $\theta_{E}$ (see [17]), where the latter can be estimated from the former, since $\theta_{E}=d\left(1-\frac{d-1}{d}\lambda_{U,E}\right)$. Recall that the various tail coefficient estimators estimate different quantities and, therefore, their values should not be compared to each other. However, a few general conclusions can be made based on Figure 7 and Figure 8. Clearly, all the studied groups possess a certain amount of tail dependence. The combinations of groups also seem to be tail dependent, although the strength of dependence is smaller. Groups G2 and G3 seem to be slightly more tail dependent than G1, which suggests that sharing industry influences tail dependence more than sharing geographical location.

The estimator of Frahm’s extremal dependence coefficient in Figure 7a,b is clearly the smallest of all the estimators, which follows its “strict” definition in (7). The dots, representing the estimates under the assumption of underlying copula being an extreme-value copula, are greater than the fully non-parametric estimators. This indicates that assuming underlying extreme-value copula might not be appropriate.

The estimator of Schmid’s and Schmidt’s tail dependence measure in Figure 7c,d is much smoother as a function of *k* than the other estimators. However, it tends to move towards 0 or 1 for very low *k*.

The estimator ${\hat{\lambda }}_{U}^{I_{2}|J_{1}}$ in Figure 8a suggests that, for all three groups, the probability of two stocks having an extremely low return given that the third stock has an extremely low return is approximately 0.2. The estimator ${\hat{\lambda }}_{U}^{I_{1}|J_{5}}$ in Figure 8d on the other hand suggests that, in all three group combinations, the largest company is heavily affected if the remaining five stocks have extremely low returns. For group combinations G1 + G3 and G2 + G3, the estimated tail coefficient is, in fact, equal to 1.

The values of ${\hat{\lambda }}_{U,E}$ and ${\hat{\theta }}_{E}$ are presented in Table 4. One can notice that these measures also suggest that groups G2 and G3 are slightly more tail dependent than G1, or, in other words, they likely contain less independent components (see [18]).

## Figures and Tables

**Table 1:** Overview of multivariate tail coefficients and their properties.

Tail Coefficient	Section	Properties
Frahm’s extremal dependence coefficient	Section 3.1	$\left(T_{1}\right)$, $\left(T_{3}\right)$, $\left(T_{4}^{\prime }\right)$, $\left(T_{5}\right)$
$\;\epsilon_{L}\left(C_{d}\right)$, $\epsilon_{U}\left(C_{d}\right)$		+ continuity property
Li’s tail dependence parameter	Section 3.2	$\left(T_{1}\right)$, $\left(T_{4}\right)$, $\left(T_{5}\right)$
$\;\lambda_{L}^{I_{h}|J_{d-h}}\left(C_{d}\right)$, $\lambda_{U}^{I_{h}|J_{d-h}}\left(C_{d}\right)$		+ continuity property
		$\left(T_{3}\right)$ (restricted sense)
Schmid’s and Schmidt’s tail dependence measure	Section 3.3	$\left(T_{1}\right)$, $\left(T_{3}\right)$, $\left(T_{4}^{\prime }\right)$
$\;\lambda_{L,S}\left(C_{d}\right)$, $\lambda_{U,S}\left(C_{d}\right)$		+ continuity property
our proposal: $\lambda_{U,S}^{*}\left(C_{d}\right)$	Section 3.3	$\left(T_{1}\right)$, $\left(T_{3}\right)$, $\left(T_{4}^{\prime }\right)$, $\left(T_{5}\right)$
		+ continuity property
Tail dependence of extreme-value copulas	Section 3.4	$\left(T_{1}\right)$, $\left(T_{2}\right)$, $\left(T_{3}\right)$, $\left(T_{4}^{\prime }\right)$
$\;\lambda_{U,E}\left(C_{d}\right)$		
Tail dependence using subvectors	Section 3.5	$\left(T_{1}\right)$, $\left(T_{2}\right)$, $\left(T_{3}\right)$, $\left(T_{5}\right)$
$\;\mu_{L}\left(C_{d}\right)$, $\mu_{U}\left(C_{d}\right)$		$\left(T_{4}\right)$ (under extra conditions on the weights)

**Table 2:** Illustrative examples: overview of tail coefficient values. NAp = Not Applicable, NAv = Not Available.

Tail Coefficient		Example/Copula			
Name	Notation	4	5	6	7
		FGM	Cuadras-Augé	Clayton	Gumbel-Hougaard
Frahm’s extremal dependence coefficients	$\epsilon_{L}\left(C_{d}\right)$	0	$\left\{\begin{array}{cc} 1 & \mathrm{if}\;\theta =1\\ 0 & \mathrm{if}\;\theta \in [0,1) \end{array}\right.$	(40)	0
	$\epsilon_{U}\left(C_{d}\right)$	0	$\left\{\begin{array}{cc} 1 & \mathrm{if}\;\theta =1\\ \theta /(d-(d-1)\theta ) & \mathrm{if}\;\theta \in [0,1) \end{array}\right.$	0	(43)
			$\lim_{d\to \infty }\theta /\left(d-\left(d-1\right)\theta \right)=0$		
Li’s tail dependence parameters	$\lambda_{L}^{I_{h}|J_{d-h}}\left(C_{d}\right)$	0	$\left\{\begin{array}{cc} 1 & \mathrm{if}\;\theta =1\\ 0 & if\;\theta \in [0,1) \end{array}\right.$	${\left(\left(d-h\right)/d\right)}^{1/\theta }$	0
				$\lim_{d\to \infty }{\left(\left(d-h\right)/d\right)}^{1/\theta }=0$	
	$\lambda_{U}^{I_{h}|J_{d-h}}\left(C_{d}\right)$	0	$\left\{\begin{array}{cc} 1 & \mathrm{if}\;\theta =1\\ \left(36\right) & \mathrm{if}\;\theta \in [0,1) \end{array}\right.$	0	$\left\{\begin{array}{cc} 1 & \mathrm{if}\;\theta =1\\ \left(44\right) & \mathrm{if}\;\theta \in [0,1) \end{array}\right.$
Schmid’s and Schmidt’s tail dependence measures	$\lambda_{L,S}\left(C_{d}\right)$	0	$\left\{\begin{array}{cc} 1 & \mathrm{if}\;\theta =1\\ 0 & \mathrm{if}\;\theta \in [0,1) \end{array}\right.$	NAv	NAv
	$\lambda_{U,S}\left(C_{d}\right)$	0	θ	NAv	NAv
our proposal	$\lambda_{U,S}^{*}\left(C_{d}\right)$	0	θ	NAv	NAv
tail dependence extreme-value copulas	$\lambda_{U,E}\left(C_{d}\right)$	NAp	θ	NAp	$\left(d-d^{1/\theta }\right)/\left(d-1\right)$
					$\lim_{d\to \infty }\left(d-d^{1/\theta }\right)/\left(d-1\right)$
					=1

**Table 3:** List of selected stocks for the analysis.

	Company Name	Industry	Country	Market Capitalization [bil. EUR]
Group 1 (G1) (German stocks)	Bayer	Pharmaceutics	Germany	48.31
	BMW	Automotive	Germany	27.81
	Deutsche Post	Courier	Germany	28.02
Group 2 (G2) (Financial stocks)	BBVA	Financial	Spain	19.39
	BNP Paribas	Financial	France	33.23
	Generali	Financial	Italy	18.41
Group 3 (G3) Energetics stocks)	Enel	Energetics	Italy	63.51
	ENGIE	Energetics	France	24.22
	Iberdrola	Energetics	Spain	53.75

**Table 4:** Estimated tail coefficients for extreme-value copulas.

	G1	G2	G3	G1 + G2	G1 + G3	G2 + G3
${\hat{\lambda }}_{U,E}$	0.50	0.63	0.58	0.61	0.57	0.64
${\hat{\theta }}_{E}$	2	1.74	1.84	2.95	3.15	2.8
